# Stable self-assembled oral metformin-bridged nanocochleates against hepatocellular carcinoma

**DOI:** 10.1007/s13346-024-01724-5

**Published:** 2024-11-13

**Authors:** Mohamed G. El-Melegy, Amal H. El-Kamel, Radwa A. Mehanna, Ahmed Gaballah, Hoda M. Eltaher

**Affiliations:** 1 Department of Pharmaceutics, Faculty of Pharmacy, Alexandria University, Alexandria, 21521 Egypt; 2 Medical Physiology Department, Faculty of Medicine, Alexandria University, Alexandria, Egypt; 3 Center of Excellence for Research in Regenerative Medicine and Applications CERRMA, Faculty of Medicine, Alexandria University, Alexandria, Egypt; 4 Microbiology Department, Medical Research Institute, Alexandria University, Alexandria, 21561 Egypt

**Keywords:** Metformin-bridged nanocochleates, Ex-vivo permeation, Caco-2 transport, HepG2, Oral bioavailability, Pharmacokinetics

## Abstract

Despite its established anti-diabetic activity, Metformin hydrochloride (MET) has been repurposed for the management of hepatocellular carcinoma (HCC). Owing to MET high aqueous solubility and poor oral permeability, a novel nanoplatform is sought to overcome the current challenges of traditional formulations. In this study, we developed MET-bridged nanocochleates (MET-CO) using a direct bridging method followed by optimization and assessment using various in-vitro and in-vivo pharmacokinetic methods. The optimized nanocochleates MET-CO_DCP_ 19, containing dicetyl phosphate (DCP), displayed uniform snail-shaped nano-rolls measuring 136.41 ± 2.11 nm with a PDI of 0.241 ± 0.005 and a highly negative ζ-potential of -61.93 ± 2.57 mV. With an impressive MET encochleation efficiency (> 75%), MET-CO_DCP_ 19 exhibited a controlled biphasic release profile, with minimal initial burst followed by prolonged release for 24 h. Importantly, they showed significant MET permeation in both in-vitro Caco-2 and ex-vivo intestinal models compared to non-DCP containing formula or MET solution. The in-vivo oral bioavailability study demonstrated pronounced improvements in the pharmacokinetic parameters with a 5.5 relative bioavailability compared to MET solution. Notably, a significant reduction in IC_50_ values in HepG2 cells after 24 h of treatment was observed. Furthermore, the optimized formulation showed a significant downregulation of anti-apoptotic and cancer stemness genes, with 12- and 2-fold lower expression compared to MET solution. These promising results highlight the efficacy of the novel MET-bridged nanocochleates as a stable nanoplatform for enhancing the oral bioavailability of MET and boosting its anticancer potential against HCC.

**Graphical Abstract:**

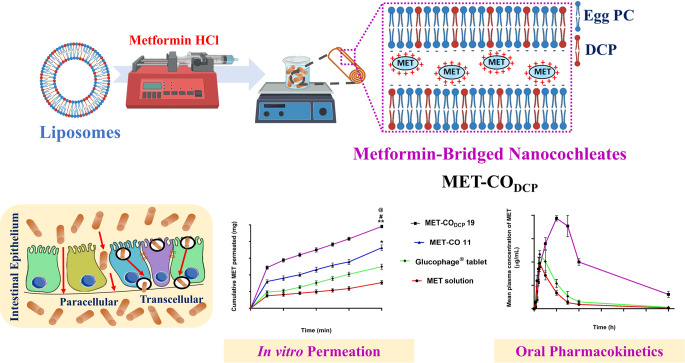

**Supplementary Information:**

The online version contains supplementary material available at 10.1007/s13346-024-01724-5.

## Introduction

Liver cancer is a global health challenge that is ranked as the sixth most common cancer and the third leading cause of cancer-related mortality worldwide, as estimated by the World Health Organization Global Cancer Observatory in 2020. By 2025, there will be an estimated incidence of > 1 million cases [1]. Siegel et al. listed liver cancer as the fifth leading cause of cancer death in 2021 in the United States [2]. Hepatocellular carcinoma (HCC) constitutes a substantial health burden, being responsible for 90% of primary liver cancers [3]. In addition to the financial cost and detrimental side effects, the application of traditional chemotherapeutics does not meaningfully prolong the survival rate nor improve the quality of life for patients diagnosed with advanced HCC. Therefore, it is necessary to identify effective therapeutic strategies for the treatment of HCC.

Metformin HCl (1,1-dimethyl biguanide hydrochloride) is one of the first-line oral anti-diabetic medicines for the treatment of type II diabetes. Interestingly, studies have widely reported that MET exerts critical inhibitory effects on the growth of various tumors including liver cancer [4, 5] with an effective role for HCC immunotherapy [6]. Unlike the inaccessibility and high hepatotoxicity of many chemotherapeutic agents, MET is a relatively inexpensive, available, and safe drug candidate.

Diabetes may critically predispose individuals to a relative risk of developing HCC. Epidemiological studies have recently revealed that treatment of diabetic patients with MET is associated with a 50% risk reduction in HCC incidence [7]. A growing body of evidence indicates that the antiproliferative effects of MET in HCC cells are predominantly regulated through activation of the adenosine monophosphate-activated protein kinase (AMPK)-dependent pathway [6, 8, 9]. Several biological mechanisms have been reported that MET probably plays a direct anti-tumor role by inhibiting HCC proliferation through induction of G1/G0 phase arrest of the cell cycle, p21CIP and p27KIP expression, and downregulation of cyclin D1 expression [10]. Moreover, MET-induced apoptosis might be triggered in HCC cells via signaling cascades, such as the AMPK and p38 mitogen-activated protein kinase [11]. In addition, a low concentration of MET can induce p53-dependent cell senescence of liver cancer cells by stimulating the AMPK pathway [12].

Regarding the physicochemical properties of MET, it is a highly hydrophilic drug (300 mg/mL) that has a completely cationic charge (> 99.9%) at physiological pH. Consequently, it has limited passive diffusion across the upper small intestine [13]. As a result, the clinical applications of MET are greatly hindered due to its poor permeability and relatively low bioavailability (50–60%) as well as the short biological half-life (t_1/2_ ~ 0.9–2.6 h) [14]. MET is classified as a Class III drug according to the Biopharmaceutics Classification System (BCS) [13]. These challenges often result in high dosing frequency to maintain effective plasma concentrations. This, in turn, reduces patient compliance and increases the incidence of gastrointestinal (GI) intolerance and weight loss. Additionally, chronic administration of MET may lead to a rare incidence of lactic acidosis, mainly in diabetic patients with comorbid conditions [15]. Given these perspectives, it is essential to develop effective nanocarriers for MET that have superior entrapment efficiency and prolonged shelf-life stability. This can ultimately enhance the overall oral permeability and maximize the anticancer efficacy of MET.

Lipid-based nanocarriers have been extensively investigated owing to their structural resemblance to the cell membrane. Trials to formulate MET in liposomal formulations including cationic niosomes, ethosomes or liposomes have been proposed for intravaginal, topical or intravenous administration [16–19]. However, clinical applications of liposomes to improve the oral absorption of water-soluble drugs remain a challenging obstacle, mostly due to their poor mechanical stability, including leakage, low drug-loading efficiency, instability after lyophilization resulting in drug leakage upon long-term storage, and limited penetration across the intestinal barrier [20]. Recently, nanocochleates have emerged as a valuable lipid-based delivery platform for enhancing the bioavailability and therapeutic activity of different drug categories, including positively charged hydrophilic drugs that were often claimed to be “challenging to deliver orally” [21, 22]. Nanocochleates are stable cigar-like spiral rolls, composed of an interaction between negatively charged phospholipids and positively charged multi-cationic metal ions or drug molecules acting as the inter-bilayer bridges [22–24]. When a bridging agent binds with the anionic phospholipid head groups of one bilayer and that of the opposite bilayer, the internal aqueous core of small unilamellar vesicles (SUVs) is excluded, resulting in lipid bilayer fusion into supramolecular self-assemblies of rolled sheets. The inimitable structure of nanocochleates is highlighted by spiral lipid sheets, in which a large, continuous, and planar phospholipid bilayer sheet is coiled around an initial point of folding with little interior aqueous space [25].

Being comprised of a series of solid rigid layers, drug molecules within the interior of nanocochleates remain relatively encochleated; namely entrapped, although the outer layers may be exposed to harsh environmental conditions [22, 23]. As an example, the utilization of water-soluble cationic tobramycin as the bridging agent in developing nanometer-sized cochleates, established upon negatively charged dioleoyl phosphatidylserine (DOPS)-based liposomes, displayed an improvement in its antibacterial activity [26]. Another example of utilizing positively charged Amikacin as the bridging agent with or without Ca^2+^, was likewise reported [27].

In this study, we have developed a framework to enhance the intestinal permeability of MET and improve its shelf-life stability. For the first time, MET-bridged nanocochleates were pre-formulated and thoroughly evaluated using a wide range of in-vitro characterization techniques. Our findings demonstrate that these nanocochleates significantly enhance oral permeation, as evidenced by in-vitro cell culture, ex-vivo rat intestinal model, and in-vivo oral pharmacokinetics study in rats. Furthermore, we conducted an in-depth evaluation of the in-vitro anticancer activity of these nanocochleates in the human liver cancer cell line, HepG2, focusing on cytotoxicity, cellular uptake, and gene expression.

## Materials and methods

### Materials

Metformin hydrochloride (MET) was a gift sample from Alexandria Co. for Pharmaceuticals & Chemical Industries (Alexandria, Egypt). Lipoid^®^ E80 (Egg phospholipids with 80% phosphatidylcholine (PC)) was a kind gift from Lipoid AG (Ludwigshafen, Germany). Cholesterol (Ch) and dicetyl phosphate (DCP) were purchased from Sigma-Aldrich (St. Louis, Mo, USA). Glucophage^®^ tablets, 500 mg (Brand product of Metformin HCl, Minapharm Pharmaceuticals under License of Merck Sante France (MERCK SERONO), Cairo, Egypt). Dulbecco’s Modified Eagle’s Medium (DMEM) high glucose, heat-inactivated fetal bovine serum (FBS), streptomycin/penicillin, Hank’s Balanced Salt Solution (HBSS), and other cell culture materials were purchased from Lonza Verviers SPRL (Belgium). 3-(4,5-dimethylthiazol-2-yl)-2,5-diphenyltetrazolium bromide (MTT) was purchased from SERVA Electrophoresis GmbH (Germany). Rhodamine-B was obtained from Sigma-Aldrich Merck (Germany). TRIzol^®^ Reagent and Hoechst 33,342 were purchased from Invitrogen, Thermo Fisher Scientific (Waltham, MA, USA). All the primers were purchased from Willowfort^®^ Co. (UK). Phenytoin was obtained as a gift sample from Amryia Pharmaceutical Co., Egypt. HPLC grade acetonitrile, methanol, and DMSO were purchased from Fischer Scientific (Loughborough, UK). All other used chemicals, solvents, and reagents were of analytical grade. Human cancer cell lines (Hepatocellular carcinoma cell line, HepG2, and Colorectal adenocarcinoma cell line, Caco-2) were obtained from the Center of Excellence for Research in Regenerative Medicine and its Applications (CERRMA), Faculty of Medicine, Alexandria University.

### MET-bridged nanocochleates (MET-CO)

#### Thin film hydration

MET-bridged nanocochleates were formulated by the direct bridging technique from blank conventional liposomal precursors prepared using a thin film hydration method [28]. Briefly, 70 mg of natural phospholipid (PL) (Lipoid^®^ E80) and 30 mg of Ch were dissolved in 5 mL of 100% chloroform in a round-bottomed flask by gentle swirling. The obtained organic solution was evaporated under a vacuum using a rotary evaporator (BÜCHI 461 Corporation, Switzerland) supplied with an oil-free vacuum pump (Rocker^®^, Model Rocker 801, Taiwan) to obtain a thin film. Trace solvents were removed by placing the flask under a vacuum for 15–20 min. The dried lipid film was then hydrated with 5 mL of phosphate buffer (0.1 M, pH 7.4) containing the predetermined dissolved amount of MET as shown in (Table S1). The dispersion was stirred for 30 min in a water bath at a constant temperature above the chain melting temperature (T_m_) of Lipoid^®^ E80 (40 ± 2 ℃). To attain nanocochleates, the resulting suspension was ultra-sonicated using a Sonoplus probe sonicator (BANDELIN electronic, Germany) operated at 70% amplitude and pulsed for 10 min (60 s-cycle) in an ice bath. Blank liposomes were prepared using the same procedure devoid of MET.

For the preparation of MET-CO using the trapping technique, 400 µL of the predetermined amount of MET dissolved in phosphate buffer, pH 7.4, was injected dropwise at a constant rate at 100 µL/min using a syringe pump (Model NE-4000, KF Technology Srl, Italy) into the prepared blank liposomes dispersion under constant magnetic stirring of 1000 rpm for 30 min (JP Selecta, Multimatic-9-N, Spain).

#### Ethanol injection

MET-CO formulations were developed using the direct bridging technique from blank conventional liposomal precursors prepared using an ethanol injection method [29]. First, 70 mg of Lipoid^®^ E80 and 30 mg of Ch, with or without the predetermined amount of DCP (Table S1), were dissolved in 2 mL of absolute ethanol and heated up to 40 ℃. Then, the dissolved mixture was rapidly injected through a 23G syringe into 5 mL of phosphate buffer (0.1 M, pH 7.4) containing MET under constant magnetic stirring (1000 rpm). The system was subjected to evaporation under vacuum for 10–15 min at 40 ± 2 ℃ to remove any residual ethanol. Afterward, it was further stirred (1000 rpm) for 1 h at room temperature. Blank liposomes were prepared using the same procedure without MET.

On the other hand, for the formulation of MET-CO using the trapping technique, blank liposomes with a final volume of 4.6 mL were prepared following the above-mentioned procedure and the subsequent steps were performed the same as described in section ‎Thin film hydration.

### In-vitro characterization

#### Particle attributes

The prepared formulations of MET-CO were subjected to mean particle size (PS), polydispersity index (PDI), and ζ-potential (ZP) analyses by dynamic light scattering (DLS) at a scattering angle of 173° using a Malvern Zetasizer^®^ (Nano ZS90, Malvern Instruments, Malvern, UK) at 25 °C. The formulations were diluted with filtered de-ionized water (1:100).

#### Product yield, drug loading, and % entrapment efficiency

Freshly prepared nanocochleates were centrifuged at 20,000 rpm for 30 min at 4 °C to separate nanoparticles from the supernatant. Pellets containing nanocochleates were resuspended in deionized water and freeze-dried using a Cryodos-50 lyophilizer (Telstar, SA, Terrassa, Spain).

Entrapment efficiency was determined using the ultrafiltration technique [24]. MET-bridged nanocochleates were added to Vivaspin^®^ centrifugal tubes (MWCO 100,000 Da, Sartorius Lab Instruments, Germany) and centrifuged using a cooling centrifuge (Sigma 3–30 K, Germany) at 6000 rpm for 15 min at 4 °C. The clear filtrates were separated, and the free unentrapped drug was measured by an Agilent Technologies-Cary 60 UV spectrophotometer (CA, USA) at λ_max_ 232 nm. The entrapment efficiency (EE %), drug loading (DL), and overall product yield (%) were calculated as detailed previously [24].

#### Morphological examination

Selected formulations were investigated for morphology using scanning electron microscopy (SEM, JSM-IT200; JEOL, Tokyo, Japan) and transmission electron microscopy (TEM, JEM-2100 F; JEOL, Tokyo, Japan) respectively. For SEM, a droplet of the sample suspension in deionized water was mounted on an aluminum stub, vacuum-dried, and sputter-coated with gold. Subsequently, the samples were scanned at an acceleration voltage of 20 kV. For TEM, thin films of freshly prepared samples diluted with filtered deionized water (1:4) were obtained by placing a sample drop onto carbon-coated copper grids. Samples were stained with an aqueous solution of uranyl acetate (1% w/v) for 30 s. TEM operated at an accelerating voltage of 80 kV.

#### Solid-state properties

Thermograms of MET, freeze-dried selected formulations, and their respective physical mixtures were recorded using Differential scanning calorimetry (DSC) (PerkinElmer Inc, Shelton, CT, USA). Briefly, 5 mg was hermetically sealed in an aluminum pan and heated at a constant rate of 10 °C/min from 25 °C to 400 °C.

Fourier-transform infrared spectrometry (FTIR) analyses of MET, PL, Ch, DCP, and selected freeze-dried formulations, and their respective physical mixtures, were performed using an FTIR spectrometer (PerkinElmer Inc, Shelton, CT, USA). Pellets were prepared by finely grinding the samples, then mixing them with pure crystalline KBr powder at a ratio of 1:100, then finally compressed into discs. The FTIR spectrum was recorded between 4000–450 cm^− 1^.

#### In-vitro MET release

The dialysis bag diffusion method [30] was used to study the release profiles of MET. Samples of the selected formulations or free drug solution equivalent to 20 mg of MET were individually placed into dialysis bags (MWCO 12,000–14,000 Da, Visking^®^, SERVA, Germany). After that, the dialysis bag was immersed in 10 mL of phosphate-buffered saline (PBS, pH 7.4) and kept under shaking conditions of 100 rpm at 37 ± 0.5 ℃. Aliquots of 0.1 mL at predetermined time intervals were withdrawn and promptly replaced with fresh pre-warmed medium. The samples were analyzed spectrophotometrically at λ_max_ 232 nm, and the cumulative percent of drug released was plotted. All data were fitted in DDSolver, an add-in program for Microsoft Excel, for modeling and comparison of drug release profiles [31].

#### Stability studies

To investigate short-term stability, selected dispersions were observed after 6 h of preparation and daily over 1 week to reveal any fluctuations in MET content. During this period, formulations were kept at 4 ± 2 °C at 25% relative humidity (RH). For storage stability, dispersions of selected formulations were stored either at 4 ± 2 °C (25% RH) or as freeze-dried powders, without any cryoprotectant, in a desiccator containing CaCl_2_ at both 25 ± 2℃ (> 5% RH) and 4 ± 2 °C (25% RH). Reconstitution of lyophilized samples was conducted in phosphate buffer, pH 7.4 at room temperature. The formulations were investigated in terms of changes in mean PS, PDI, ZP, and %EE after 1, 3, and 6 months in addition to SEM and TEM.

### Permeation studies

#### In-vitro permeation

Caco-2 cells were seeded on PET filter inserts (ThinCert™ insert, Greiner Bio-One, Germany) with a culture surface area of 113.1 mm^2^; pore size of 0.4 μm in the apical chambers, at a seeding density of approximately 10^5^ cells/well. The inserts were placed onto 12-well Transwell^®^ cell-culture plates containing 2 mL of medium in the basolateral chamber. The integrity of the monolayer culture was monitored using inverted light microscopy. A Hoechst 33,342 staining assay, performed with a confocal laser scanning microscope (CLSM) (Leica^®^ Microsystems Inc. Model DMi8, Wetzlar, Germany), was used to confirm the integrity of the Caco2 cell monolayer.

Permeation studies across the Caco2 monolayers were performed from the apical to the basolateral direction in Hank’s Balanced Salt Solution (HBSS) of adjusted pH 6.8 at 37 °C. Selected formulations or MET solution in phosphate buffer, pH 7.4 (equivalent to 10 mg MET), were pipetted into the apical side of the chambers. At predetermined time intervals, 0.2 mL aliquots were withdrawn from the basolateral side of the chambers and replaced with an equal volume of fresh pre-warmed HBSS. Experiments were performed in triplicate. MET permeated to the basolateral side was analyzed using a reported validated HPLC method developed by Choi et al. [32] with slight modifications (Supplementary Data S1). The apparent permeability coefficient (P_app_) values of MET and the optimized formulations were calculated (Supplementary Data S2) as detailed previously [24].

#### Ex-vivo intestinal permeation

Ex-vivo permeation studies were carried out using the non-everted intestinal sac model [33]. A total of 20 male Wistar albino rats (weighing 200 ± 20 g) were used in the study. Experiments were performed according to the approval and ethical guidelines of the Institutional Animal Care and Use Committee (IACUC) of Alexandria University (AU 062021321196), along with the UK Animals (Scientific Procedures) Act, 1986 and the European Community Guidelines for Animal Experiments (EU Directive 2010/63/EU). The intestinal excision protocol was adopted from our previously published work [24]. Each sac was filled with selected formulations or MET solution (phosphate buffer, pH 7.4) equivalent to 10 mg of MET powder or crushed Glucophage^®^ tablets. The two sides of the intestine were tied firmly with a thread. Each non-everted intestinal sac was soaked in 10 mL of the diffusion medium (Ringer’s solution, pH 6.8) to achieve sink conditions. The entire system was maintained at 37^°^C in a shaking water bath operated at 100 rpm and well aerated with 5% CO_2_ and 95% O_2_ (10–15 bubbles/min). Samples (1 mL) were withdrawn from outside of the sac after predetermined time intervals and simultaneously compensated with fresh pre-warmed medium. Samples were filtered through 0.22 μm PTFE syringe filters (Chromtech^®^, UK) followed by analysis by HPLC.‎ The study was performed in triplicate. The cumulative amount of MET permeated was plotted [34] and then P_app_ values of the samples were calculated.

### In-vivo pharmacokinetics

An in-vivo oral pharmacokinetics study was performed on adult male Wistar albino rats weighing 200 ± 20 g. The study followed the guidelines for animal handling and treatment approved by the Institutional Animal Care and Use Committee (IACUC) of Alexandria University (AU 062021321196). Healthy animals were housed in standard metal cages at a temperature of 23 ± 2 °C and a relative humidity of 55%, with a 10-h light/14-h dark cycle. They were supplied with standard rat chow and water. Prior to the day of the experiment, the rats fasted overnight for 16 h and had free access to water ad libitum. A total of 15 rats were randomly allocated into 3 groups (*n* = 5). Each group received a single oral dose equivalent to MET (200 mg/kg), as previously reported [35]. The first group received MET solution, while the other two groups received crushed Glucophage tablets (in phosphate buffer, pH 7.4) or MET-CO_DCP_ 19 dispersion, respectively. During the first 6 h, the animals were allowed to drink water freely, with restricted food access. After that, the rats were supplied with food. A volume of 0.5 mL of blood was rapidly collected from the retro-orbital plexus at predetermined time intervals using EDTA tubes.

Blood samples were promptly centrifuged (4000 rpm, 10 min) and the separated plasma was stored at -20 °C. MET concentration in rat plasma was analyzed using the previously mentioned HPLC method with the use of phenytoin as an internal standard [36]. Briefly, a phenytoin standard solution (9 µg/mL) was added at a 1:1 ratio. The spiked samples were deproteinized with acetonitrile at a 1:2 ratio, vortexed for 20 s, and centrifuged (10,000 rpm, 10 min at 20^°^C) for complete precipitation of plasma proteins. The clear supernatant was withdrawn and filtered through a 0.22 μm PTFE syringe filter and then injected into the HPLC column (Supplementary Data S1).

The estimation of pharmacokinetic parameters based on a non-compartmental pharmacokinetic model [37] was performed using PKSolver, an add-in program for Microsoft Excel, for analyzing pharmacokinetic data [38]. The total areas under the plasma concentration-time curve (AUC _0→24 h_) and (AUC _0→∞_) were determined using the linear trapezoidal method. The peak plasma concentration (C_max_) and the time to reach peak plasma concentration (T_max_) were determined from graphs.

### Anti-cancer activity

#### Cell proliferation

The cytotoxic effects of MET were evaluated using the MTT assay [39]. HepG2 cells were seeded into 96-well plates (5 × 10^3^ cells/well) and incubated for 24 h before adding MET solution or selected formulations at different concentrations (5, 10, 20, 30, 40, 50, 60, and 70 mM) for 24 and 48 h. Afterward, 50 µL of MTT solution (5 mg/mL MTT in PBS) was added to each well and the cells were incubated for 4 h in the dark at 37 °C and 5% CO_2_. Then, the culture media were removed, and 150 µL of DMSO was added to each well followed by gentle shaking of the plates on an orbital shaker for 10 min to dissolve the formazan crystals. Cell viability was estimated by measuring the absorbance at λ_max_ 570 nm (A_570 nm_) using an automated ELISA microplate reader (BioTek^®^ Instruments, VT, USA). Results were expressed as a percentage of cell viability relative to control untreated cells. The 50% growth inhibitory concentration (IC_50_) was calculated from a plotted dose-response curve using non-linear regression analysis in GraphPad Prism (version 7.04).

#### Cellular uptake

Rhodamine-B was used as a water-soluble red fluorescent dye to visualize the uptake of MET-bridged nanocochleates. All test formulations were fluorescently labeled with aqueous rhodamine-B dye at a final concentration of 1 µg/mL. HepG2 cells were seeded over coverslips in 6-well plates and incubated for 1 h with the labeled formulations equivalent to the IC_50_ of MET solution. The cells were then fixed, washed with PBS, and observed using a confocal laser microscope (Leica microsystems, DMi8, Wetzlar, Germany). Identical fields of each image were analyzed to generate 3D surface plots. The brightness values were recorded and expressed as mean red fluorescence intensity using Image J software (version 1.52a).

#### Gene expression

HepG2 cells were cultured in 12-well plates (5 × 10^5^ cells/well) for 24 h. Cells were then treated with the IC_50_ (24 h-treatment) of MET solution, the optimized formulation, and its blank counterpart for 24 h. The specific primer pairs used for amplification of Bcl-2, NANOG target genes, and GAPDH were designed using NCBI Primer Blast online software and synthesized via Willowfort^®^ Co., UK as previously detailed [24]. Quantitative Real-time PCR (qRT-PCR) was performed using a Stratagene Mx3000P qPCR system (Agilent, Massy, France). Relative quantities of each target gene were calculated using the 2^–ΔΔCt^ method [39].

### Statistical analyses

All experiments were conducted in triplicate unless otherwise stated, and the data were expressed as the mean ± standard deviation (SD). Statistical differences were determined using an unpaired Student’s t-test or one-way ANOVA followed by Tukey’s multiple comparisons using GraphPad Prism (Version 7.04, San Diego, CA, USA). The level of significance was set at (*p* ≤ 0.05).

## Results and discussion

### Formulation of MET-CO

Lipoid^®^ E80 was selected as a natural negatively-charged phospholipid, imparting a negative zeta potential to the liposomal surface under physiological pH condition [40]. Cholesterol was included to act as a membrane stabilizer for the formed SUVs in the dispersion [41]. Blank liposomes prepared via thin film hydration are usually subjected to probe sonication for the preparation of SUVs. The sonication step is often associated with reduced yield, low entrapment efficiency, degradation of phospholipids and sensitive drugs, elimination of large metal contaminants from the probe tip, and the presence of multi-lamellar vesicles (MLVs) along with SUVs [20].

As shown in Table 1, the MET-CO formulations prepared using the direct bridging technique (DB) had larger nanometric sizes compared to the blank liposomes (B1), with the MET-CO 4 formula (highest MET loading 100 mg) having the largest average size and highest PDI value. Likewise, upon increasing MET loading in formulations developed using the trapping technique (MET-CO 5, 6, and 7) the average size of the nanocochleates increased. However, their mean particle sizes were slightly smaller than those prepared by the direct bridging technique. Interestingly, the particle size and PDI values point to the unique spiral-shaped rolls of MET-bridged nanocochleates, distinct from the typical vesicular structure. Such varied results could be ascribed to the nature and strength of electrostatic interaction forces occurring between the cationic drug and anionic lipid components. Unlike the slow and weak interaction between MET and liposomal vesicles in the trapping technique, direct bridging would provide stronger interplay between the charged moieties of MET and lipid excipients, directly developing the nano-self-assembly of MET-CO formulations. This explanation could appear in the findings of relatively higher size and PDI values of MET-CO formulations prepared by the direct bridging technique than those prepared by the trapping technique.

Likewise, zeta potential measurements might indicate that most of the positive MET molecules in the direct bridging technique were found inside the interior roll’s layers rather than bound at its surface as in the trapping technique. As a result, this would contribute to developing electrostatically powerful MET-bridged nanocochleates whilst maintaining their negative surface charge. Furthermore, another suggestion has been proposed that MET was considered a constituting material of the nano-self-assembly structure rather than a simple entrapped moiety which could explain the relative superiority of entrapment efficiency, payload and yield in the direct bridging technique over that of the trapping technique.

Unlike the thin film hydration method, the ethanol injection method produces stable nano-sized MET-bridged nanocochleates with a homogenous size distribution. This is achieved by slowly adding dissolved ethanolic lipophilic matrix into an aqueous buffer while stirring magnetically. This process converts the nanocochleates’ precursors from multilamellar vesicles (MLVs) to small unilamellar vesicles (SUVs). In this study, the average nano-size of MET-bridged nanocochleates produced prepared by the ethanol injection/direct bridging method (MET-CO 9, 10, and 11) increased with the amount of MET added. A similar observation was reported when larger quantities of Amikacin were employed in the development of Amikacin-bridged cochleates [27]. The PDI values slightly increased from 0.151 to 0.201 compared to 0.241 to 0.306 for thin film hydration counterparts (Table 1). However, the PDI values of MET-CO were still relatively higher compared to the blank vesicular counterpart prepared via ethanol injection (B8). This can be explained in light of the morphological structure of MET-bridged nanocochleates, which resemble elongated cigar-like assemblies with a small radius. As a result, the measured hydrodynamic diameters are more widely dispersed compared to spherical liposomes, which have a narrow polydispersity [24, 42].

A slight reduction in the negative zeta potential values indicated the formation of MET-bridged nanocochleates, reaching − 40.64 ± 1.02 mV for MET-CO 11 with the maximum MET loading. This was accompanied by improvement in %EE and DL compared to counterparts prepared using thin-film hydration/direct bridging. On the other hand, MET-CO 12, 13, and 14 prepared using the trapping technique showed slightly smaller nanometric size and PDI values compared to the corresponding ethanol injection/direct bridging formulations. However, the zeta potential of MET-CO 14, was found to be -44.65 ± 2.01 mV. This was further reflected in lower %EE and %yield (41.52 ± 2.38% of encochleated MET and 70.35 ± 0.94% yield). This indicates the better inclusion of positive MET in the nanocochleates’ core of MET-CO 11, resulting in higher %EE and % yield (54.68 ± 2.49 and 78.5 ± 1.27 respectively). Accordingly, MET-CO 11 was selected as the optimal formula based on the optimum ionic coupling between negatively charged egg PC and positively charged bridged MET molecules. Notably, these results indicated the preferable industrial scalability of MET-bridged nanocochleates prepared by the direct bridging technique rather than the conventional trapping technique.

The reported high entrapment efficiencies of Trans-Resveratrol in Ca^2+^-bridged cochleates [24] could not be replicated with the freely water-soluble MET. In this context, one strategy to optimize the entrapment efficiency was to use a negatively charged surfactant DCP. This surfactant enhances the overall negative zeta potential, which helps with the binding of MET within the nanocochleate’s spiral sheets. MET-CO_DCP_ formulations with 10 mg of DCP and 100 mg of MET (MET-CO_DCP_ 16 and MET-CO_DCP_ 17) had a slightly larger nanometric size compared to the MET-CO 11 formula. However, MET-CO_DCP_ 17 had a higher negative zeta potential (-58.16 ± 2.38 mV). Additionally, MET-CO_DCP_ 16, prepared using the direct bridging technique, had a higher %EE value (60.15 ± 1.34%).

To further improve the efficient encochleation of MET molecules, 20 mg of DCP was investigated. Increasing DCP content might decrease the pH of the colloidal suspension [43] which could affect the zeta potential of the MET-bridged nanocochleates and enhance the inclusion of MET. It was observed that MET-CO_DCP_ 19 and MET-CO_DCP_ 20, prepared by the direct bridging and trapping techniques, respectively, possessed relatively small particle sizes and PDI values close to the MET-CO 11. The slight increase in the particle size of DCP-containing nanocochleates could be attributed to the substantial recruitment of more MET molecules to be tethered inside the nanocochleates’ spiral sheets, indicating a better encapsulation of MET that triggered the formation of more self-assembled rolled layers. The encapsulation efficiency was remarkably increased, reaching 76.83 ± 1.68% in the MET-CO_DCP_ 19 formula. Similar to %EE, DL, and the product yield of the MET-CO_DCP_ 19 was significantly higher than other MET-CO formulations.

To conclude, MET loading as the bridging agent for nanocochleates development appears to be core factor for the nano-self-assembly of MET-CO formulations with 100 mg loading being the optimal load for coherent nanocochleates with highly negative zeta potential and highest entrapment efficiency. This is complemented using the efficient ethanol injection/direct bridging method that produces stable nano-sized MET-bridged nanocochleates with a homogenous size distribution. Finally, the inclusion of negatively charged surfactant DCP enhances the overall negative zeta potential contributing to the integration of MET within the assembled spiral sheets. MET-CO_DCP_ 19, prepared by ethanol injection/direct bridging with 100 mg MET loading and 20 mg DCP, was selected as the optimal formula for MET-bridged nanocochleates. It possessed a desirable nanometric size of 136.41 ± 2.11 nm, a reasonable PDI of 0.241 ± 0.005, and a relatively high negative zeta potential of -61.93 ± 2.57 mV. To our knowledge, this present study is the first to report the development of self-assembled MET-bridged nanocochleates: tubular rolls of MET-associated egg PC-DCP using the reproducible cutting-edge direct bridging technique of MET molecules.

**Table 1:** In-vitro characterization of MET-bridged nanocochleates and their respective blank liposomes

Method	Technique	Formula code	Average size (nm)	PDI	ζ-potential (mV)	% Entrapment Efficiency	Drug loading (mg/g)	% Yield
Thin Film Hydration		**B1**	191.48 ± 6.76	0.198 ± 0.01	-46.31 ± 1.25	-------	-------	-------
	DB	MET-CO 2	226.3 ± 5.61	0.241 ± 0.008	-45.02 ± 1.18	15.6 ± 2.02	78 ± 10.1	67.12 ± 0.69
		MET-CO 3	254.68 ± 7.24	0.285 ± 0.013	-40.97 ± 3.21	28.53 ± 1.32	213.98 ± 9.9	64.35 ± 1.82
		MET-CO 4	281.25 ± 4.12	0.306 ± 0.017	-36.43 ± 2.87	40.22 ± 1.75	402.2 ± 17.5	68.59 ± 1.04
	T	MET-CO 5	201.59 ± 4.62	0.228 ± 0.019	-45.69 ± 1.09	9.82 ± 3.78	49.1 ± 18.9	65.03 ± 2.15
		MET-CO 6	232.3 ± 6.06	0.259 ± 0.011	-42.53 ± 2.31	20.14 ± 1.21	151.05 ± 9.07	60.24 ± 1.61
		MET-CO 7	258.11 ± 5.9	0.286 ± 0.012	-39.78 ± 1.62	33.86 ± 2.29	338.6 ± 22.9	67.52 ± 0.98
Ethanol Injection		**B8**	104.02 ± 1.15	0.116 ± 0.005	-50.47 ± 2.72	-------	-------	-------
	DB	MET-CO 9	108.13 ± 3.19	0.151 ± 0.004	-48.11 ± 2.32	21.55 ± 1.62	107.75 ± 8.1	74.65 ± 2.49
		MET-CO 10	115.26 ± 2.53	0.172 ± 0.009	-45.42 ± 0.55	35.4 ± 2.83	265.5 ± 21.23	71.23 ± 2.01
		**MET-CO 11**	**128.61 ± 1.45**	**0.201 ± 0.003**	**-40.64 ± 1.02**	**54.86 ± 2.49**	**548.6 ± 24.9**	**78.5 ± 1.27**
	T	MET-CO 12	105.74 ± 1.9	0.139 ± 0.008	-49.15 ± 1.98	16.29 ± 1.91	81.45 ± 9.55	71.64 ± 1.39
		MET-CO 13	110.52 ± 3.05	0.157 ± 0.005	-47.06 ± 1.59	29.73 ± 2.61	222.98 ± 19.58	69.18 ± 1.11
		MET-CO 14	117.04 ± 2.31	0.173 ± 0.006	-44.65 ± 2.01	41.52 ± 2.38	415.2 ± 23.8	70.35 ± 0.94
		**B15**	104.63 ± 1.74	0.12 ± 0.003	-61.57 ± 3.21	-------	-------	-------
	DB	MET-CO_DCP_ 16	130.17 ± 3.09	0.213 ± 0.011	-52.4 ± 1.59	60.15 ± 1.34	546.82 ± 12.18	79.66 ± 0.54
	T	MET-CO_DCP_ 17	115.97 ± 2.73	0.181 ± 0.009	-58.16 ± 2.38	46.03 ± 1.27	418.45 ± 11.55	74.43 ± 1.11
		**B18**	105.12 ± 1.01	0.127 ± 0.006	-83.45 ± 1.91	-------	-------	-------
	**DB**	**MET-CO**_**DCP**_ **19**	**136.41 ± 2.11**	**0.241 ± 0.005**	**-61.93 ± 2.57**	**76.83 ± 1.68**	**640.25 ± 14**	**91.14 ± 1.53**
	T	MET-CO_DCP_ 20	120.15 ± 2.06	0.202 ± 0.007	-75.42 ± 1.8	55.1 ± 2.37	459.17 ± 19.75	80.69 ± 1.24

*Abbreviations* PDI, Polydispersity Index; DB, Direct Bridging; T, Trapping. Results are expressed as mean ± SD, (*n* = 3)

**Fig. 1:**
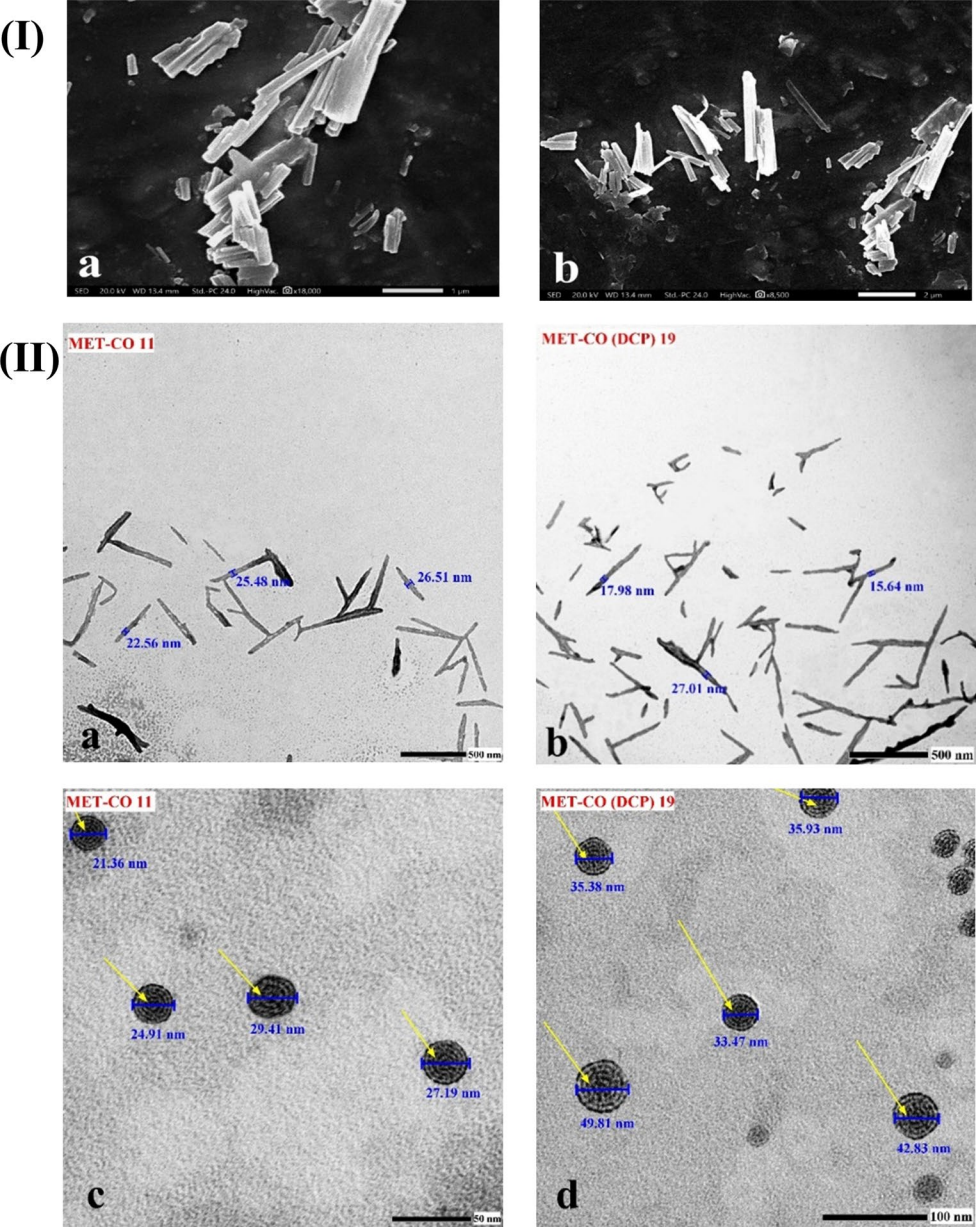
Morphological examination of MET-bridged nanocochleates. **(I)** SEM images of (a) MET-CO 11 and (b) MET-CO_DCP_ 19. Scale bars (a) 1 μm and (b) 2 μm. **(II)** TEM images of (a) MET-CO 11, (b) MET-CO_DCP_ 19, (c) MET-CO 11, and (d) MET-CO_DCP_ 19. Scale bars (a, b) 500 nm, (c) 50 nm and (d) 100 nm. Yellow arrows point out the snail-like structure of nanocochleates

SEM investigation was conducted to analyze changes in surface morphology. As depicted in Fig. 1 (I) a & b, elongated cylindrical structures of MET-bridged nanocochleates were observed. The rolls adhered to each other and overlapped after vacuum drying inside the SEM machine. The SEM images confirm the distinct surface morphology of the MET-bridged nanocochleates, which differed significantly from the conventional spherical vesicles in terms of the length-to-width aspect ratio.

TEM revealed that formulations exhibited slightly smaller mean diameters compared to their zeta sizer measurements. This was attributed to the change in the geometry of the nanocochleates cylinders from the ideal spherical shape used in dynamic light scattering (DLS) analysis of particle size. As shown in Fig. 1(II) a & b, TEM confirmed the presence of long cylinders for MET-CO 11 and MET-CO_DCP_ 19 formulations with an average length of approximately 470.48 ± 90.09 nm and 507.25 ± 93.1 nm, respectively. Using Image J software, the average widths of the MET-CO 11 and MET-CO_DCP_ 19 rolls were determined to be 25.72 ± 2.1 nm and 39.48 ± 4.33 nm, respectively. This finding supports the improved encapsulation efficiency of the bridging drug, MET molecules, within the lipid bilayers of the MET-CO_DCP_ 19 spiral sheets. Additionally, both MET-CO 11 and MET-CO_DCP_ 19 exhibited unique helical structures, as indicated by the pointed arrows in Fig. 1(II) c & d. Interestingly, these structures contained small internal aqueous spaces. The average length of the inter-rolling layer spaces within MET-CO 11 and MET-CO_DCP_ 19 was measured to be 1.97 ± 0.05 nm and 1.34 ± 0.02 nm, respectively, indicating minimal interior aqueous spaces. Collectively, dynamic light scattering, entrapment efficiency, and microscopy confirm the successful development of MET-bridged nanocochleates in a manner similar to our previously reported drug-loaded Ca^2+^-bridged nanocochleates [24].

**Fig. 2:**
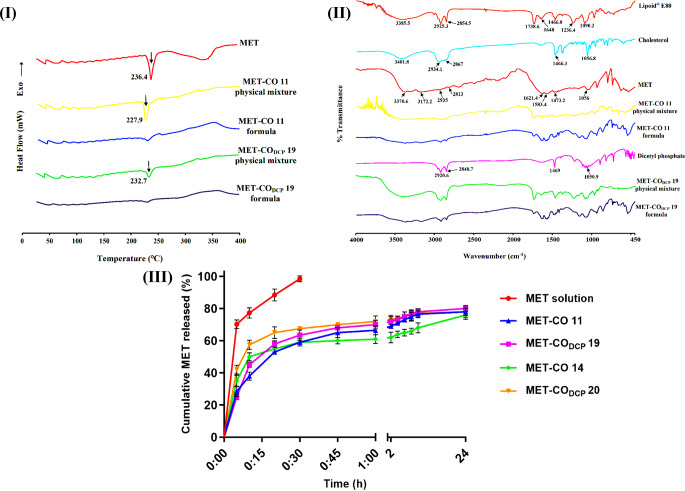
In-vitro characterization of MET-bridged nanocochleates. (**I**) DSC thermograms and (**II**) FTIR spectra of MET, MET-CO 11 physical mixture, MET-CO 11 formula, MET-CO_DCP_ 19 physical mixture, and MET-CO_DCP_ 19 formula along with FTIR spectra of egg PC (Lipoid^®^ E80), cholesterol and dicetyl phosphate (DCP). (**III**) In-vitro release profile of MET from drug solution (in phosphate buffer, pH 7.4), MET-CO 11, MET-CO 14, MET-CO_DCP_ 19, and MET-CO_DCP_ 20 at 100 rpm and 37 °C using dialysis bag method. Data are expressed as mean ± SD, (*n* = 3)

The DSC thermograms of MET, MET-bridged nanocochleates, and their corresponding physical mixtures are displayed in Fig. 2(I). It was observed that MET displayed a single sharp endothermic peak at 236.4 °C, which corresponds to the melting point of MET [44]. The sharpness of the drug peak indicates the high purity of MET and suggests predominantly crystalline behavior. The drug peaks were detected in the thermograms of the physical mixtures, indicating no physicochemical interaction between MET and the other constituent components. In contrast, the MET-CO 11 and MET-CO_DCP_ 19 thermograms did not exhibit an endothermal peak of MET, suggesting the incorporation of MET in an amorphous or molecularly dispersed state within the MET-bridged nanocochleates. Consequently, it is expected that encochleation would enhance MET diffusion and membrane permeability.

FTIR spectroscopy was conducted to elucidate the intermolecular interaction between MET and excipients. For comparative purposes, egg PC, cholesterol, and DCP were included in the analysis, and all spectrum curves of MET-CO 11, MET-CO_DCP_ 19, and their respective physical mixtures are illustrated in Fig. 2(II). The FTIR spectrum of egg PC (Lipoid^®^ E80) displayed characteristic peaks at 3386 cm^− 1^ (broad absorption band of -OH stretching), 2925 and 2855 cm^− 1^ (C-H stretching of the fatty acid backbone), 1739 cm^− 1^ (C = O stretching of the ester group), 1648 cm^− 1^ (C = C stretching of aliphatic chain), 1467 cm^− 1^ (C-H bending of hydrocarbon chain), 1236 cm^− 1^ (C-N stretching band) and 1090 cm^− 1^ (C-O stretching) were detected. The cholesterol spectrum revealed an intense and broad absorption peak approximately at 3402 cm^− 1^ due to hydroxyl group stretching. Also, it showed a typical band between 2867 and 2934 cm^− 1^ representing stretching vibrations of -CH_2_ and -CH_3_ groups.

Additionally, bands at 1466 and 1057 cm^− 1^ were revealed due to vibrations of C-H bending and C-O stretching, respectively. The FTIR spectrum of DCP showed a characteristic band between 2849 and 2921 cm^− 1^ due to C-H stretching of the hydrocarbon chain as well as absorption peaks at 1469 cm^− 1^ and 1051 cm^− 1^ referring to C-H bending and C-O stretching, respectively. Similar FTIR spectra of lipid components were previously reported [24, 45].

The spectrum of MET exhibited intense and broad absorption peaks of -NH_2_ and -NH stretching at 3371 cm^− 1^ and 3172 cm^− 1^, respectively. The absorption peaks at 2935 and 2813 cm^− 1^ corresponded to C-H stretching of -CH_3_ groups and the bands at 1621 cm^− 1^ and 1583 cm^− 1^ indicated C = N stretching. The bending band at 1565 cm^− 1^ was attributed to the deformations of N-H. The bands at 1473 cm^− 1^ and 1056 cm^− 1^ referred to vibrations of C-H bending and C-N stretching, respectively. An identical FTIR spectrum of MET was previously reported [46].

The FTIR spectra of the physical mixtures of either MET-CO 11 or MET-CO_DCP_ 19 showed combination peaks for the comprising components, confirming the lack of chemical interactions. On the other hand, the spectra of MET-CO 11 and MET-CO_DCP_ 19 displayed the above-mentioned components’ peaks but masked the major peaks relating to -OH stretching of lipid components as well as -NH_2_ and -NH stretching of MET along with more obviously concealed their stated bands, particularly in MET-CO_DCP_ 19 formula. Hence, these observations indicate a significant ionic interaction between positively charged amino groups of bridged MET and negatively charged functional groups of the lipophilic ingredients, egg PC and DCP, upon formulation, as illustrated in the supplementary data (Fig. S1). In conclusion, these findings confirm the integration of MET molecules within the nanocochleates assemblies, demonstrating strong ionic forces that bind MET to the bilayer lipid’s spiral sheets. This supports the efficient bridging of MET within the coiled crystal matrix.

### In-vitro MET release

MET solution (phosphate buffer, pH 7.4) exhibited a higher release rate compared to the MET-CO formulations Fig. 2(III). Approximately 70% and 99% of the cumulative MET was released after 5 and 45 min, respectively. This can be attributed to the high hydrophilicity of MET in PBS medium, pH 7.4. In contrast, MET-CO 14 and MET-CO_DCP_ 20 demonstrated a relatively higher initial burst release, with 35% and 42% of the initial MET content released after 5 min, and nearly 76% and 78% released over 24 h, respectively. On the other hand, MET-CO 11 and MET-CO_DCP_ 19 exhibited burst release values of 28.5% and 25%, respectively. This suggests minimal MET adsorption on the surface of the nanocochleates, indicating the efficient bridging of MET molecules within the inner continuous spiral layers’ core of the nanocochleates compared to the counterparts prepared using the trapping technique.

The release of MET from MET-CO_DCP_ 19 reached approximately 45% and 63% after 10 and 30 min, respectively, compared to 58% and 68%, respectively, for MET-CO_DCP_ 20. The controlled release phase could be initiated by the slow unfolding of the folded MET-bridged nanocochleates’ matrix, facilitating the diffusion of MET into the dissolution medium. The release profiles of MET-CO 11 and MET-CO 14 showed no significant difference, with 59% of MET released after 30 min. On the other hand, DCP as a surfactant in both MET-CO_DCP_ 19 and MET-CO_DCP_ 20 played a key role in further enhancing MET release rate as both formulations have been encochleated to superior MET extents than other non-DCP formulations (MET-CO 11 and MET-CO 14).

Overall, the release of MET from MET-bridged nanocochleates exhibited a biphasic profile, with an initial release of 25% in 5 min, followed by sustained release of 79% for up to 24 h from the MET-CO_DCP_ 19 formula, compared to a burst release of 70% and 98.5% released within 30 min from the MET solution. The sustained release behavior of the MET-CO_DCP_ 19 formula can be beneficial for drugs with high aqueous solubility, such as MET, as it allows for the slow introduction of the drug to gastrointestinal fluids for absorption, thereby enhancing its oral bioavailability [47]. These findings indicate that the solid, rigid structure of MET-bridged nanocochleates successfully retains MET within the multilayered rolls, preventing rapid leakage, and gradually releasing MET into the medium.

To elucidate the release mechanism of MET from the MET-CO formulations, the release profiles were fitted into different kinetic models; First order, Korsmeyer-Peppas, and Weibull models (Table S2). The first-order model best described the release pattern of MET solution, MET-CO 11, and MET-CO_DCP_ 19 with the highest correlation coefficients and T_lag_ values corresponding to -0.177, -0.115, and − 0.043 h, respectively. It is noteworthy that F_max_ values of the First order model, were found to be 73.51 and 74.96 for MET-CO 11 and MET-CO_DCP_ 19, respectively, and 99.98 for MET solution. On the other hand, the Korsmeyer–Peppas model represented the best-fitting release models for MET-CO 14 and MET-CO_DCP_ 20. Furthermore, the release exponent values (n) of the Korsmeyer–Peppas model were found to be less than 0.5 (*n* < 0.15) for all tested formulations of MET-CO. Overall, these findings emphasized that the release of MET from MET-bridged nanocochleates was controlled by Fickian diffusion.

### Stability studies

Observing MET-CO 11 and MET-CO_DCP_ 19 dispersions over one week of fridge storage, %EE was found to minimally change compared to their zero-time %EE as illustrated in (Table S3). Thus, both MET-CO 11 and MET-CO_DCP_ 19 were not the only optimized MET-bridged nanocochleates in terms of loading and controlled release but also demonstrated short-term physical stability as they preserved the encochleated MET molecules inside the spiral sheets.

**Fig. 3:**
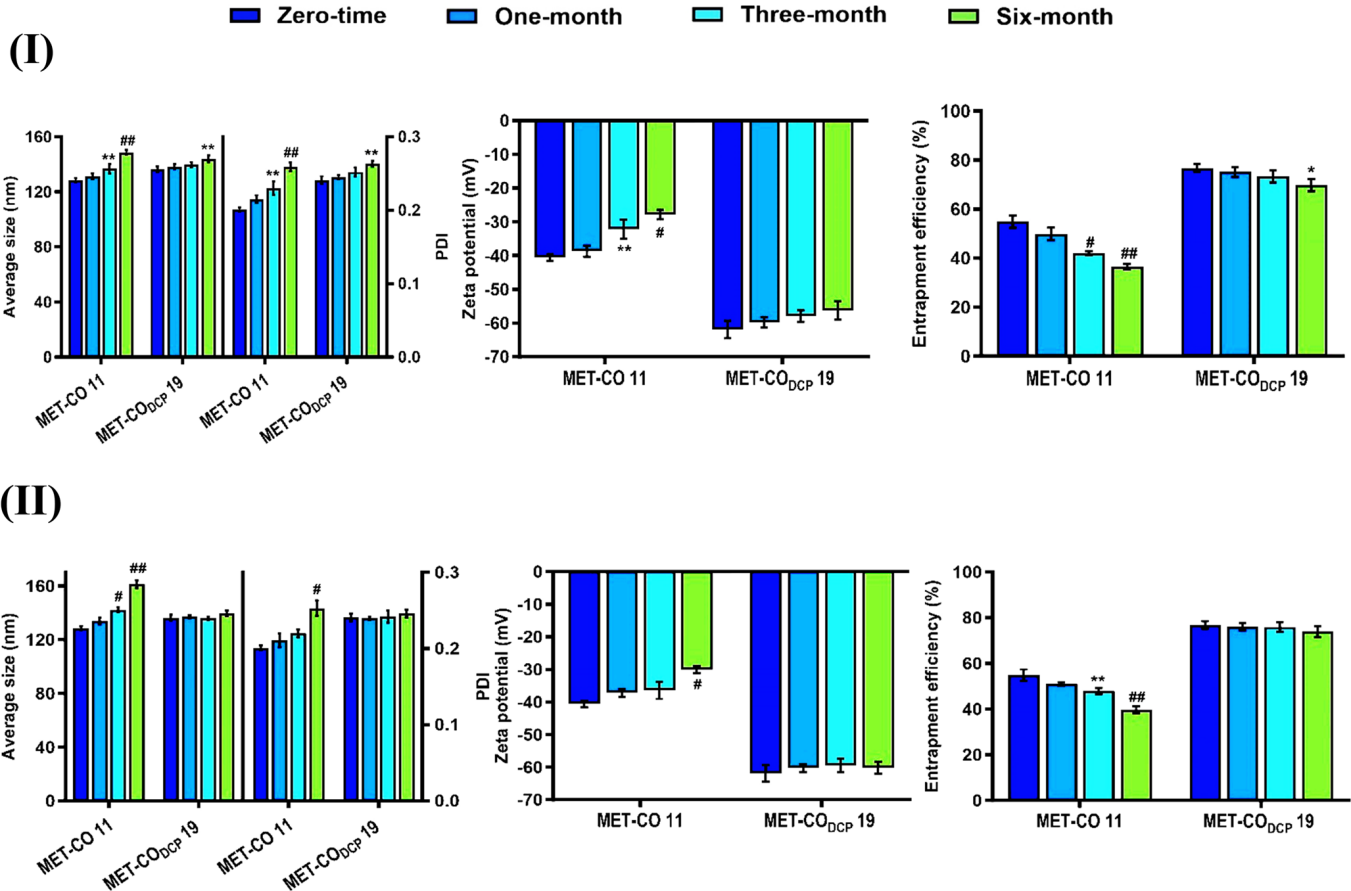
Long-term stability studies of the optimized formulations of MET-bridged nanocochleates (MET-CO 11 and MET-CO_DCP_ 19) after 6 months of storage in the form of (**I**) dispersion at 4 ± 2 ℃ and 25% relative humidity (RH) as well as (**II**) freeze-dried powder at 25 ± 2 ℃ and > 5% RH. Results are presented as mean ± SD, (*n* = 3). Data were analyzed using one-way ANOVA followed by a post-hoc test (Tukey’s). Statistical differences were significant (* *p* ≤ 0.05, ** *p* ≤ 0.01, # *p* ≤ 0.001, ## *p* ≤ 0.0001 vs. zero-time)

As demonstrated in Fig. 3 (I), MET-CO_DCP_ 19 dispersion remained relatively stable for 6 months at 4 ± 2 °C. The changes in mean particle size, PDI, and % entrapment efficiency were found to be non-significant (up to 3 months). However, after 6 months, the MET-CO_DCP_ 19 showed a significant increase in size and PDI reaching 144.01 ± 2.46 nm and 0.263 ± 0.004, respectively while the % entrapment efficiency decreased to about 69.8%. No significant changes in the negative surface-charged MET-CO_DCP_ 19 were observed. On the other hand, MET-CO 11 dispersion showed a significant increase in mean particle size and PDI:136.87 ± 3.41 nm and 0.23 ± 0.009, along with an obvious decrease in the negative zeta potential reaching − 32.2 mV after 3 months. Additionally, the % entrapment efficiency of MET dropped significantly to 42%. This could be ascribed to physical instability where considerable MET molecules leaked from nanocochleates’ folded rolls into the surrounding buffer medium, indicative of the existence of moderate ionic interaction between MET and egg PC components.

As shown in Fig. 3 (II), the particle size of the reconstituted lyophilized MET-CO 11 powder increased significantly by 10.7% and 25.4% after storage at 25 ± 2 °C for 3 and 6 months, respectively. In contrast, the particle size of MET-CO_DCP_ 19 experienced insignificant changes of -0.4% and 2.3%. The increase in particle size of MET-CO 11 was accompanied by a significant increase in PDI, while MET-CO_DCP_ 19 showed a non-significant increase in polydispersity value after 6 months. These findings suggest that the lyophilized MET-CO 11 nanocochleates’ rolls uncoiled into larger vesicles, resulting in the observed increase in particle size after reconstitution. Additionally, the negative zeta potential values of MET-CO 11 decreased significantly from − 40.6 to approximately − 30 mV, whereas MET-CO_DCP_ 19 showed a negligible reduction from − 61.9 to -60.1 mV after 6 months. Furthermore, the %EE of freeze-dried MET-CO 11 decreased significantly to 39.7% after 6 months, indicating potential drug leakage upon reconstitution. In contrast, there was negligible change in MET-CO_DCP_ 19 throughout the storage period. These results highlight the superior ability of MET-CO_DCP_ 19 to retain MET within their spiral cylindrical structures during lyophilization for extended storage periods.

**Fig. 4:**
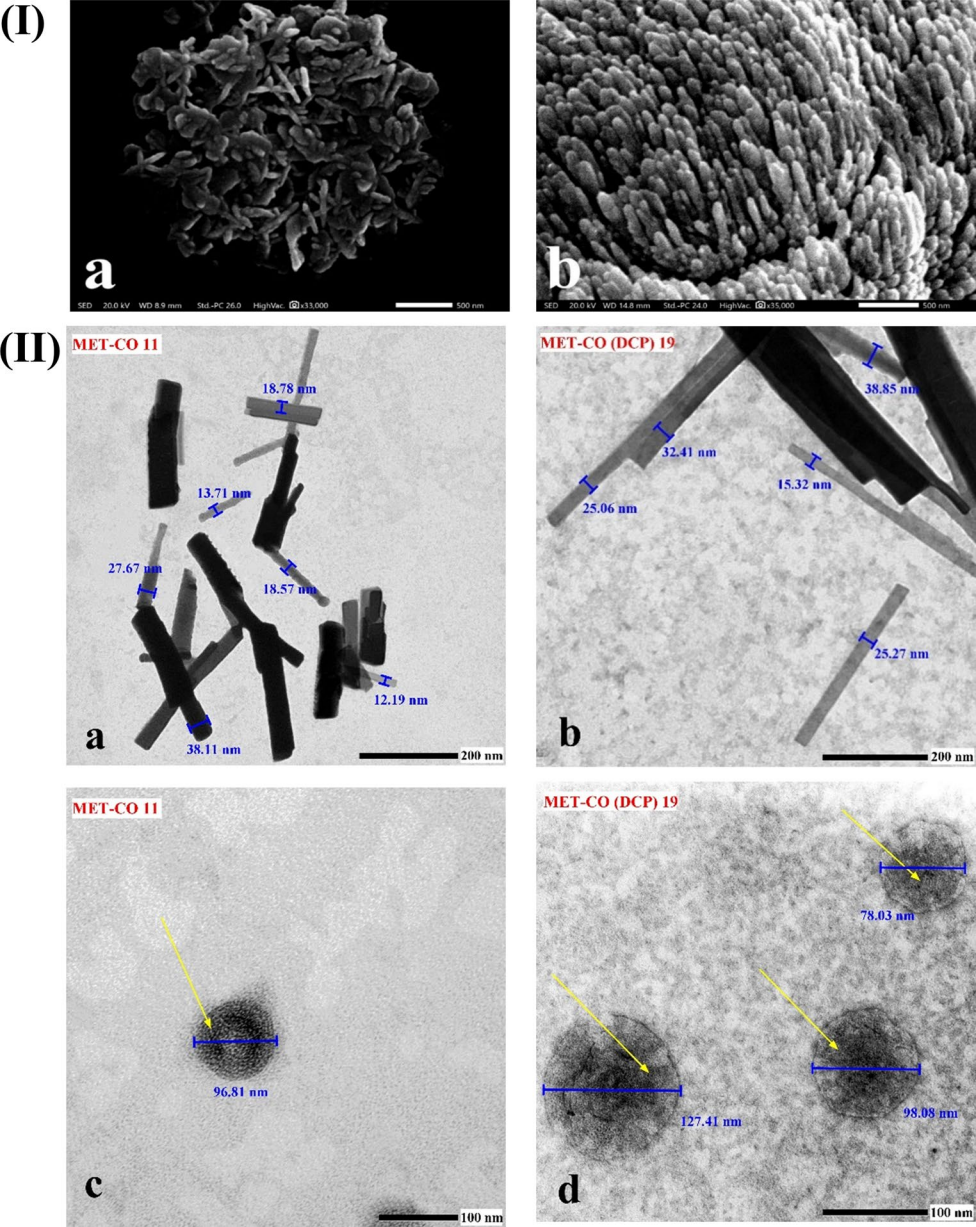
Morphological examination of MET-bridged nanocochleates after 6 months of storage at 25 ± 2 ℃ and > 5% relative humidity (RH). (**I**) SEM images of reconstituted lyophilized powders of (a) MET-CO 11 and (b) MET-CO_DCP_ 19. Scale bar 500 nm. (**II**) TEM images of reconstituted lyophilized powders of (a) MET-CO 11, (b) MET-CO_DCP_ 19, (c) MET-CO 11, and (d) MET-CO_DCP_ 19. Scale bars (a, b) 200 nm and (c, d) 100 nm. Yellow arrows indicate the spiral rolling-up structure of nanocochleates

Upon reconstitution of powders stored at 4 ± 2 °C, the particle size of MET-CO 11 nanocochleates significantly increased after 3 and 6 months, by 14.3% and 32.4% respectively, compared to 1.1% and 2.6% for MET-CO_DCP_ 19 (Fig. S2). The PDI value for MET-CO 11 increased significantly, while the negative zeta potential and %EE decreased to -27.6 mV and 36.3% respectively after 6 months. In contrast, lyophilized MET-CO_DCP_ 19 showed no significant changes in PDI, zeta potential, and %EE values throughout the storage period. These findings indicate that MET-CO_DCP_ 19 can remain physically stable under various storage conditions, as a dispersion/lyophilized powder at 4 ± 2 °C or lyophilized at room temperature for up to 6 months. SEM and TEM images (Fig. 4 (I and II)) confirmed DLS measurements, showing that freeze-dried MET-CO 11 and MET-CO_DCP_ 19 maintained their nanosized, monodispersed elongated cylinder and rolled-up structures after storage at room temperature for 6 months. However, MET-CO 11 experienced a relative increase in particle size.

In contrast to MET-CO 11, MET-CO_DCP_ 19 can be stored as a stable lyophilized powder without significant leakage of the bridging agent (MET) or damage to their unique structures. This allows for long-term storage at room temperature. The colloidal and shelf-life stabilities of MET-CO_DCP_ 19 nanocochleates are attributed to the increased negative charge of DCP nanocochleates, which triggers electrostatic stabilization and prevents fusion and aggregation of cylindrical rods during storage. Moreover, the lipid phase of the nanocochleates is arranged in a tightly packed all *trans*-configuration which has restricted mobility compared to the liquid crystalline phase [24, 48] eventually improving the mechanical stability of MET-bridged nanocochleates. Consequently, MET-CO_DCP_ 19 nanocochleates can be used as a stable free-flowing powder for oral administration in enteric-coated capsules.

### Permeation studies

The Caco-2 cell model mimicking small intestine epithelia represents an invaluable tool for investigating drug permeation [49] and is often recruited to infer oral bioavailability. Confocal microscopy images of Caco-2 cells-stained nuclei after 7 days; prior to the transport studies, revealed a dense monolayer lacking intercellular spaces confirming Caco-2 monolayer integrity (Fig. S3).

**Fig. 5:**
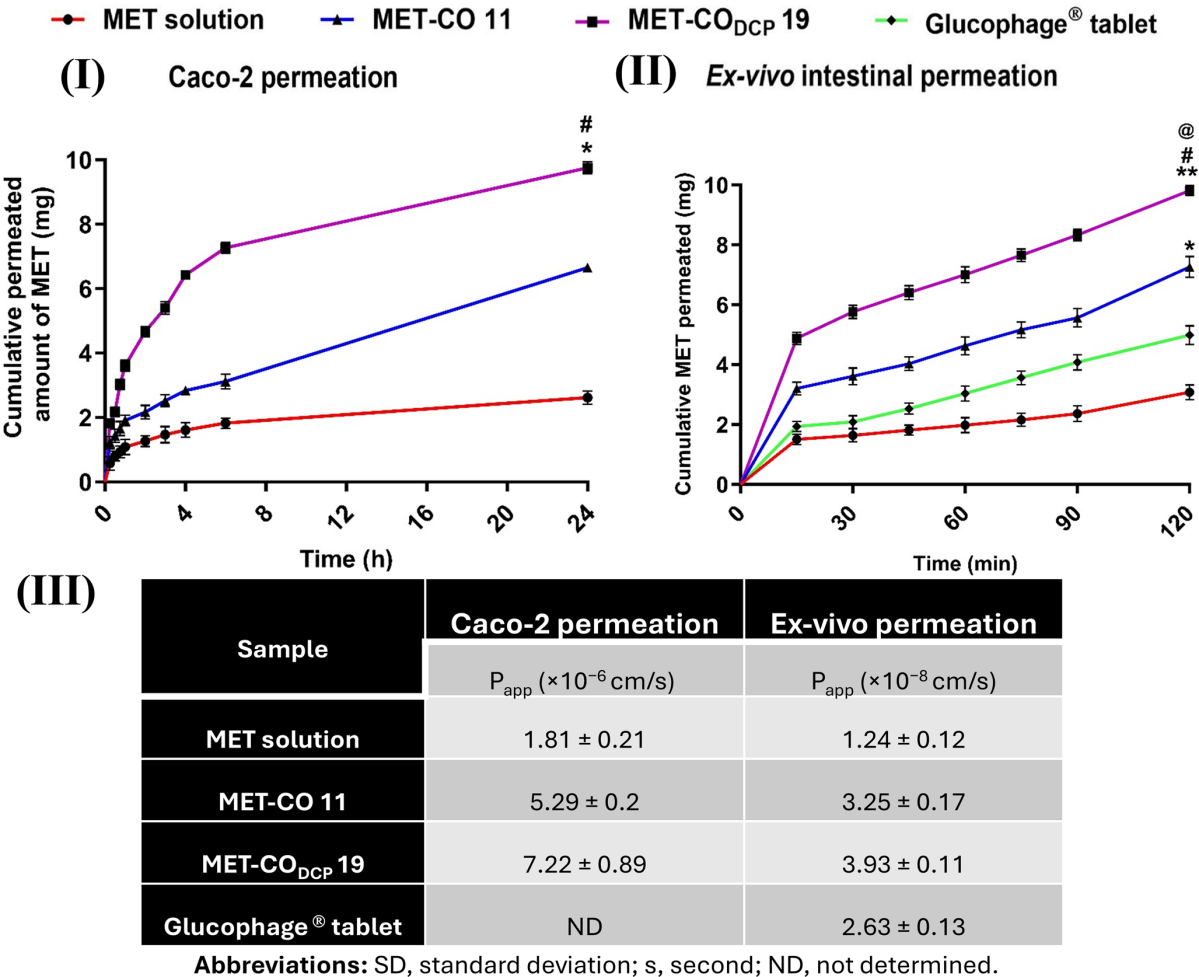
Permeation studies of MET-bridged nanocochleates. **(I)** Transport profiles of MET solution, MET-CO 11, and MET-CO_DCP_ 19 across Caco-2 monolayer. **(II)** Ex-vivo permeation of MET (equivalent to 10 mg MET) through non-everted rat intestine from MET solution, crushed Glucophage^®^ tablets (phosphate buffer, pH 7.4), MET-CO 11, or MET-CO_DCP_ 19. **(III)** Calculation of apparent permeability coefficient (P_app_) values of MET solution, crushed Glucophage^®^ tablet, MET-CO 11, or MET-CO_DCP_ 19 from Caco-2 transport and ex-vivo intestinal permeation studies. All results are expressed as mean ± SD and are statistically analyzed using unpaired Student’s t-test, (*n* = 3). Statistical differences were significant for **(I)** (* *p* ≤ 0.001 vs. MET solution and # *p* ≤ 0.05 vs. MET-CO 11) and **(II)** (* *p* ≤ 0.01, ** *p* ≤ 0.0001 vs. MET solution, @ *p* ≤ 0.0001 vs. crushed Glucophage^®^ tablet, and # *p* ≤ 0.01 vs. MET-CO 11)

MET solution, MET-CO 11, and MET-CO_DCP_ 19 were individually dispersed in the apical compartments and the cumulative amounts of MET permeated through Caco-2 monolayers were calculated at different time intervals over 24 h to delineate the transport profiles (Fig. 5 (I)). MET-CO_DCP_ 19 exhibited a markedly enhanced permeation, with a value of 9.75 ± 0.19 mg, indicating that nearly 98% of MET was successfully transported across the Caco-2 monolayer within 24 h. Conversely, MET solution and MET-CO 11 showed lower MET transport after 24 h, with 2.62 ± 0.2 and 6.65 ± 0.15 mg, respectively.

The apparent permeability coefficient (P_app_) for all the tested samples was calculated using the linear portion of the transport curves (Fig. 5 (III)). The transport efficiency of MET-CO_DCP_ 19 was 1.4- and 4-fold higher than MET-CO 11 and MET solution, respectively. As the transport experiment in this study was carried out in Caco-2 cells without Peyer’s patch model, MET-bridged nanocochleates mainly permeated through the epithelial cells. Accordingly, this finding could be attributed to the pronounced competence of MET-bridged nanocochleates to open intercellular tight junctions and paracellular pathways. Alternatively, MET-bridged nanocochleates might be uptaken via clathrin- and caveolin-mediated endocytosis [50]. A previous study reported the presence of cationic sites for the adsorption of negatively charged nanocarriers by Caco-2 cells [51]. Henceforth, a suggestion has been put forward that the higher negative surface charge of MET-CO_DCP_ 19 than that of MET-CO 11 could lead to better binding at the cationic sites on Caco-2 cells in the form of clusters. The presence of these clusters and non-specific adsorption process by Caco-2 cells might be the driving force for superior enhanced MET-CO_DCP_ 19 nanocochleates’ adsorptive endocytosis comparable to that of MET-CO 11.

Ex-vivo permeation techniques are widely employed for evaluating human intestinal absorption. Among these techniques, the non-everted intestinal sac model stands out for its simplicity, ability to utilize small drug quantities, minimal morphological damage to the intestine, and convenient sampling for quantitative analysis [33]. As shown in Fig. 5 (II), MET solution poorly permeated across the intestinal epithelium (3.08 ± 0.24 mg in 2 h). On the other hand, MET-CO 11 showed a significantly higher permeation of MET (7.26 ± 0.35 mg) compared to 4.99 ± 0.31 mg permeated from crushed Glucophage^®^ tablets after 2 h. The relatively higher permeation of MET from Glucophage^®^ tablets over MET solution could be ascribed to the coating layer of Hypromellose polymer which facilitated the penetration of MET across the intestinal epithelia [52].

For MET-CO_DCP_ 19, 5.76 ± 0.22 mg of MET permeated after 30 min compared to 1.64 ± 0.21, 2.09 ± 0.2, and 3.61 ± 0.28 mg from MET solution, crushed Glucophage^®^ tablets and MET-CO 11, respectively. Furthermore, MET permeation from MET-CO_DCP_ 19 continued to increase to 9.81 ± 0.17 mg after 2 h which was significantly higher than permeation from MET solution, crushed Glucophage^®^ tablets, or MET-CO 11 (Fig. 5 (II)). Consequently, MET-CO_DCP_ 19 accomplished superior P_app_ value than either MET solution, crushed Glucophage^®^ tablets, or MET-CO 11, as demonstrated in Fig. 5 (III), indicating outstanding permeation of these nanocochleates when also compared to findings of another reported delivery system [53]. Collectively, MET-CO_DCP_ 19 demonstrated 1.5-, 2.3- and 3.5-fold higher ex-vivo permeation of MET after 60 min compared to MET-CO 11, crushed Glucophage^®^ tablets, and MET solution, respectively.

The enhanced permeability could be ascribed to the nanocochleates’ membrane fusion capability [54] and the high tension of nanocochleates edges in which their nano-self-assembly geometry might be the driving force to interfere with cell membranes [22, 26]. Most importantly, the highly negative surface charges of MET-CO_DCP_ 19 nanocochleates suggest they will remain intact as a result of maintaining the strong ionic-tethering of MET molecules within their rolls while permeating across the intestinal epithelium. This was consistent with the estimated mechanism by which nanocochleates could enhance oral permeability by opening tight junctions of Caco-2 cells and allowing for paracellular transport. In conclusion, these data corroborated that MET-CO_DCP_ 19 nanocochleates possess an efficient potential to enhance the oral permeability of MET through the intestinal epithelia.

### In-vivo oral pharmacokinetics

The calibration curve of MET in plasma exhibited linear behavior across the range of 0.5–70 µg/mL, with a correlation coefficient (R^2^) of 0.9998. The percentage recovery ranged from 96.21 to 110.7%, while the CV% values were less than 9.5%. These findings indicate that the method’s accuracy and precision meet the guidelines set by the International Conference on Harmonization (ICH). The retention times of MET and phenytoin peaks in plasma were 4.5 min and 7.2 min, respectively, which confirms well-separated chromatographic peaks consistent with a previously reported study [55].

The newly developed nanocarrier systems are being studied in vivo using experimental animal models to monitor their fate within the organism [56]. Rats were selected due to their ability to predict drug absorption through oral administration and their similarity to the human intestinal barrier [57]. Such an investigation provides crucial data on the impact of MET-bridged nanocochleates on the oral bioavailability of the poorly permeable MET.

**Fig. 6:**
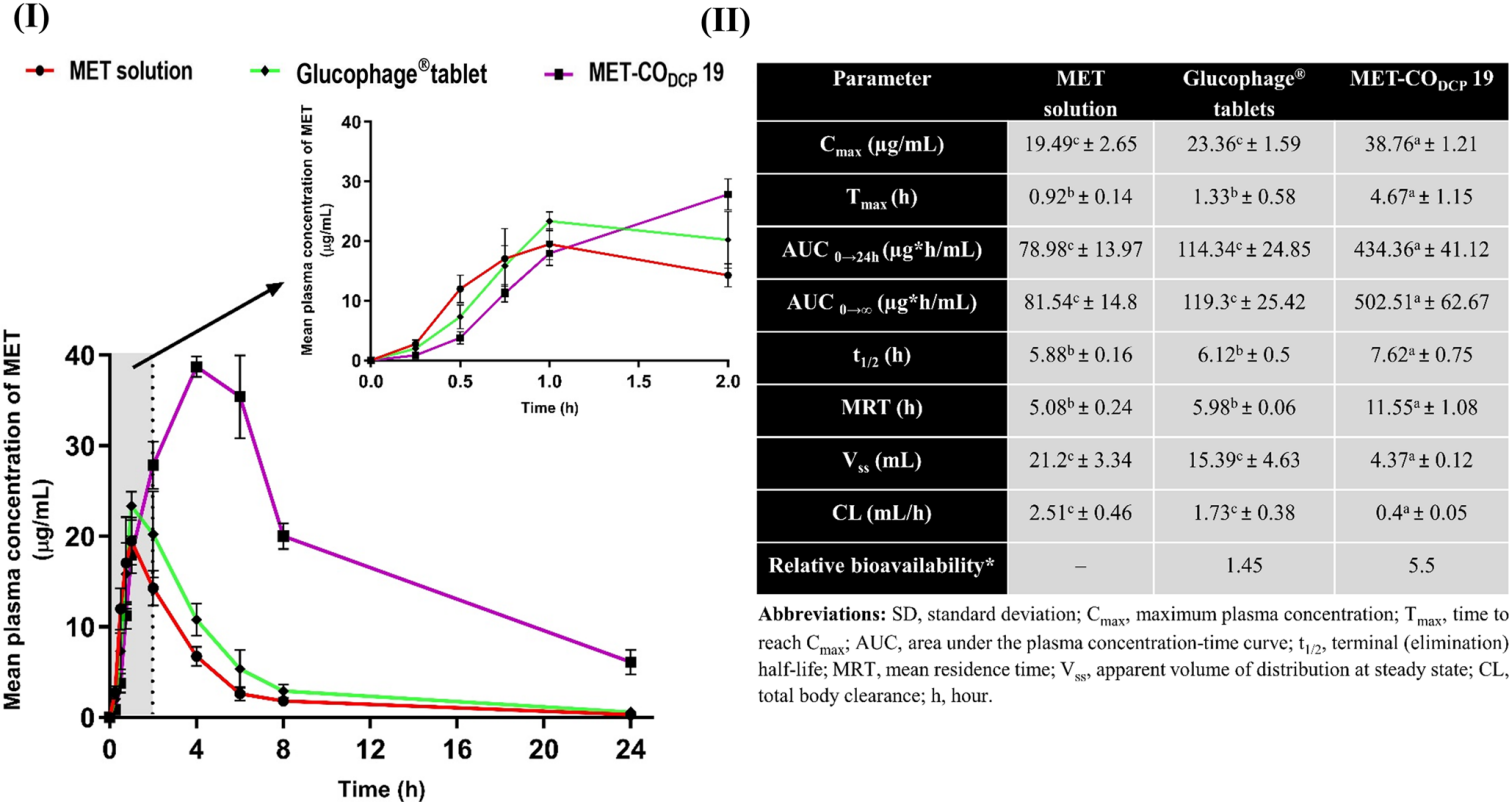
In vivo pharmacokinetics study. **(I)** Mean plasma concentration-time profile of MET after oral administration of MET solution, crushed Glucophage^®^ tablets, and MET-CO_DCP_ 19 at a single MET dose of 200 mg/kg to healthy rats. Inset represents a close-up profile of the first 2 h. **(II)** Calculated pharmacokinetic parameters of MET after oral administration of MET solution, crushed Glucophage^®^ tablets, and MET-CO_DCP_ 19 nanocochleates at a single MET dose equivalent of 200 mg/kg to healthy rats. All data are expressed as mean ± SD and statistically analyzed using one-way ANOVA followed by a post-hoc test (Tukey’s), (*n* = 5). The level of significance was set at *p* ≤ 0.05, values bearing dissimilar letters are significantly different: a > b > c. *Relative bioavailability was calculated by dividing the value of AUC _0→24 h_ of either commercial tablets or nanocochleates formula by that of MET solution

**Fig. 7:**
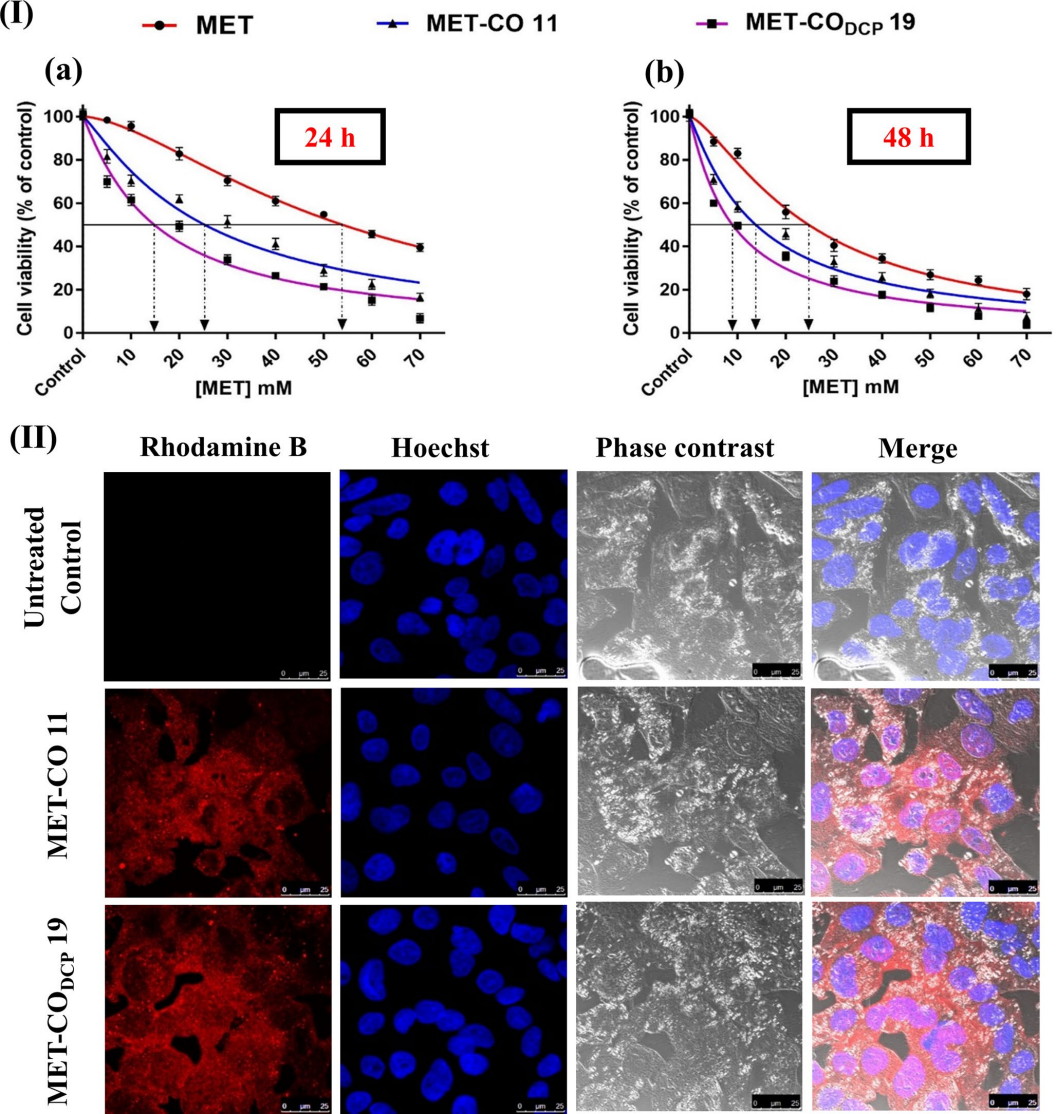
**(I)** In-vitro cytotoxicity studies of MET-bridged nanocochleates in HepG2 cell line. Cells were treated with serial concentrations of MET, MET-CO 11, MET-CO_DCP_ 19, and their respective blank formulations equivalent to [5–70] mM for (a) 24 h and (b) 48 h. Cell viability was measured by MTT assay. Data are expressed as mean ± SD, (*n* = 3) and subjected to non-linear regression for determination of IC_50_ using GraphPad Prism (Version 7.04). Black extrapolated dotted lines correspond to the IC_50_ values. **(II)** Confocal laser scanning microscope (CLSM) images showing cellular uptake of MET-CO 11 and MET-CO_DCP_ 19 in HepG2 cells after 1 h treatment with their respective IC_50_ values. Scale bar 25 μm

The mean plasma concentration-time profile was plotted after administration of a single dose of the control MET solution, Glucophage^®^ tablets, or MET-CO_DCP_ 19 equivalent to 200 mg MET/kg body weight (Fig. 6 (I)). The pharmacokinetic parameters were calculated and listed in (Fig. 6 (II)). The rate of intestinal MET absorption from the control MET solution was rapid, with a short T_max_ value of 0.92 h to achieve a C_max_ value of 19.49 ± 2.65 µg/mL (Fig. 7). Like the control MET solution, the T_max_ value of the crushed Glucophage^®^ tablet revealed an insignificant change in MET absorption with a slight increase in the C_max_ value to 23.36 ± 1.59 µg/mL. On the other hand, the oral absorption of MET from MET-CO_DCP_ 19 was significantly prolonged (*p* ≤ 0.01), reaching the C_max_ after 4.67 h. The T_max_ value of MET-CO_DCP_ 19 was found to be extended, being 3.5- and 5.1-fold longer than the crushed tablet and the control MET solution, respectively. The C_max_ value was 38.76 ± 1.21 µg/mL, which is 1.66- and 2-fold higher than that of the crushed tablet and the control MET solution, respectively.

AUC _0→t_ and AUC _0→∞_ parameters are pivotal parameters for the assessment of bioavailability [58]. As shown in Fig. 6 (II), there was an insignificant increase in the value of AUC _0→24 h_ from 78.98 ± 13.97 µg*h/mL of the control MET solution to 114.34 ± 24.85 µg*h/mL of the commercial tablet solution. On the other hand, MET-CO_DCP_ 19 nanocochleates indicated a markedly elevated value of 434.36 ± 41.12 µg*h/mL, which is about 3.8- and 5.5-fold higher than the commercial tablet solution and the control MET solution, respectively. Moreover, MET-CO_DCP_ 19 nanocochleates demonstrated a significant (*p* ≤ 0.0001) enhancement in the value of AUC _0→∞_ by 4.2- and 6.2-folds, respectively. Additionally, a significant increase in both T_1/2_ (*p* ≤ 0.05) and the mean residence time (MRT) (*p* ≤ 0.0001) was observed in MET-CO_DCP_ 19 compared to that of the tablet and the control solution. Furthermore, the time-averaged total body clearance (CL) of MET-CO_DCP_ 19 from the body decreased to a value of 0.4 mL/h which was 4.3- and 6.2-fold lower than the tablet and the control solution, respectively. Moreover, the apparent volume of distribution at steady state (V_ss_) of MET-CO_DCP_ 19 significantly decreased by 3.5- and 4.9-fold, respectively, indicating minimal biodistribution of nanocochleates throughout the body.

The lower C_max_ and AUC 0→24 h of the MET solution were mainly due to its extreme hydrophilicity, resulting in poor permeability across the intestinal epithelial cells. Similarly, commercial tablets slightly increased both C_max_ and bioavailability (AUC 0→24 h) by approximately 1.2- and 1.4-fold compared to the control solution. This variation observed in the tablet form following oral administration could be attributed to the presence of hypromellose polymer as an excipient in the powder mixture of the tablets, which may provide a negligible improvement in the calculated pharmacokinetic parameters of MET [59]. Albeit the oral absorption of MET is moderately facilitated by a saturable carrier in the commercial product, the profile of MET plasma concentration established by MET-CO_DCP_ 19 has been extended in a way different from commercial tablets and MET solution. A key reason beyond this finding could be ascribed to the presence of the hydrophobic nanocochleates’ spiral rolls resulting in the slow release of the inter-bridged MET molecules. This in turn contributed to preventing the saturation of MET transporters, extending the circulation time, and reducing its clearance.

Previously reported nanocarrier-based formulations have endeavoured to improve the oral bioavailability of MET [60, 61]. A 1.27- and 2-fold increase in the AUC _0→t_ values of MET-encapsulated borneol W/O/W composite submicron emulsion [60] and MET-loaded niosomes [61] composed of span 40 and cholesterol, as compared to MET solution, respectively. Most notably, our developed MET-CO_DCP_ 19 demonstrated significant improvement, as evidenced by a substantial increase in oral bioavailability (AUC _0→24 h_) with a 5.5-fold enhancement compared to the MET solution.

Overall, the presented pharmacokinetics results were in good agreement with the ex-vivo permeation outcomes in which a markedly enhanced permeation of MET-CO_DCP_ 19 across the intestinal barrier was observed. Boosting the oral bioavailability of MET could be ascribed to the enhanced uptake of lipid-based self-assemblies of MET-CO_DCP_ 19 by the lymphatic system in the small intestine escaping the hepatic first-pass metabolism. Another perspective could be the negatively charged surface imparted by DCP (-61.93 ± 2.57 mV), which protects against enzymatic degradation throughout the GIT. The negative surface charge promotes transcytosis of the lipid cylinders and their uptake via M-cells of Peyer’s patches in intestinal lymphatic tissues [43, 62]. Moreover, it is anticipated that the distinct self-assembly of these nanocochleates could play a fundamental role in upholding the bridged MET molecules within their interior multi-folded rolls shielding MET from the surrounding GIT environment [54]. Whereas their external layers would fuse with the epithelial membrane via their highly stressful edges and then be transported to the systemic circulation. Collectively, these capabilities could contribute to activating both transcellular and paracellular transport of MET-CO_DCP_ 19 through the intestinal barrier resulting in a prolonged half-life of MET in the bloodstream. Altogether, the advantages of the distinctive spiral structure of MET-CO_DCP_ 19 nanocochleates were combined with the beneficial effects exerted by the lipid components on the permeation across the intestinal membrane. This resulted in a significant enhancement of MET oral bioavailability.

### Anticancer activity

Cytotoxicity profiles of MET, MET-CO 11, MET-CO_DCP_ 19, and their blank counterparts against human liver cancer HepG2 cells are illustrated in Fig. 7 (I). MET-CO or the corresponding blank liposomal formulations B8 or B18 were used in the range of 16.5–232 µL/mL (dispersion/culture media) equivalent to 0–70 mM MET. Blank liposomal formulations exhibited no significant cytotoxicity against HepG2 cells similar to the controls up to 165.6 µL/mL indicative of good biocompatibility of excipients at these concentrations. Nonetheless, there were slight toxicities of blank counterparts (B8 and B18) with 90% and 84.5% viability following 24 and 48 h post-treatment, respectively at the highest scrutinized volume of blank formulations, 232 µL/mL. This could be due to the inherent anticancer activity of egg PC which was reported to be associated with its unique apoptosis-inducing properties through the generation of reactive oxygen species or its direct disturbing influence on the order of cell membrane causing fluidity and leakage [63].

MET solution, MET-CO 11, and MET-CO_DCP_ 19 showed dose- and time-dependent growth inhibitions where MET-CO_DCP_ 19 possessed a significantly higher antiproliferative activity against HepG2 cells. After 24 h incubation, the IC_50_ values of MET, MET-CO 11, and MET-CO_DCP_ 19 were found to be 53.93 ± 0.78, 25.28 ± 1.19, and 14.82 ± 0.83 mM while the IC_50_ values after 48 h, were decreased to 24.76 ± 0.6, 13.77 ± 0.69 and 8.92 ± 0.51 mM, respectively. IC_50_ values of MET solution after treatment for 24 h and 48 h were consistent with the previous report [64]. The reduction of IC_50_ values of MET-CO_DCP_ 19 to 1.7- and 3.6-fold lower than that of MET-CO 11 and MET solution, might be ascribed to the accumulation of MET via direct interaction or phagocytosis, the fusion between nanocochleates and the cancer cell membrane followed by controlled drug release [23]. MET molecules are bridged within the internal layers of solid, stable, self-assembled structures of nanocochleates. We postulate that once inside HepG2 cells, the gradient diffusion of MET triggers the unrolling of nanocochleates releasing the encochleated MET. In conclusion, MET-CO_DCP_ 19 nanocochleates demonstrated a substantial antiproliferative efficacy against HepG2 cells with a significant dose reduction.

Following the antiproliferative effects demonstrated in HepG2 cells, the cellular uptake and co-localization of labelled MET-bridged nanocochleates were investigated as shown in Fig. 7 (II). MET-CO 11 and MET-CO_DCP_ 19 demonstrated an equivalent uptake of MET in HepG2 cells after incubation for 1 h with the corresponding 24 h IC_50_. It is worth mentioning that the cellular uptake of MET-CO_DCP_ 19 was based on a lower concentration of MET (14.82 ± 0.83 mM) compared to 25.28 ± 1.19 mM for MET-CO 11.

**Fig. 8:**
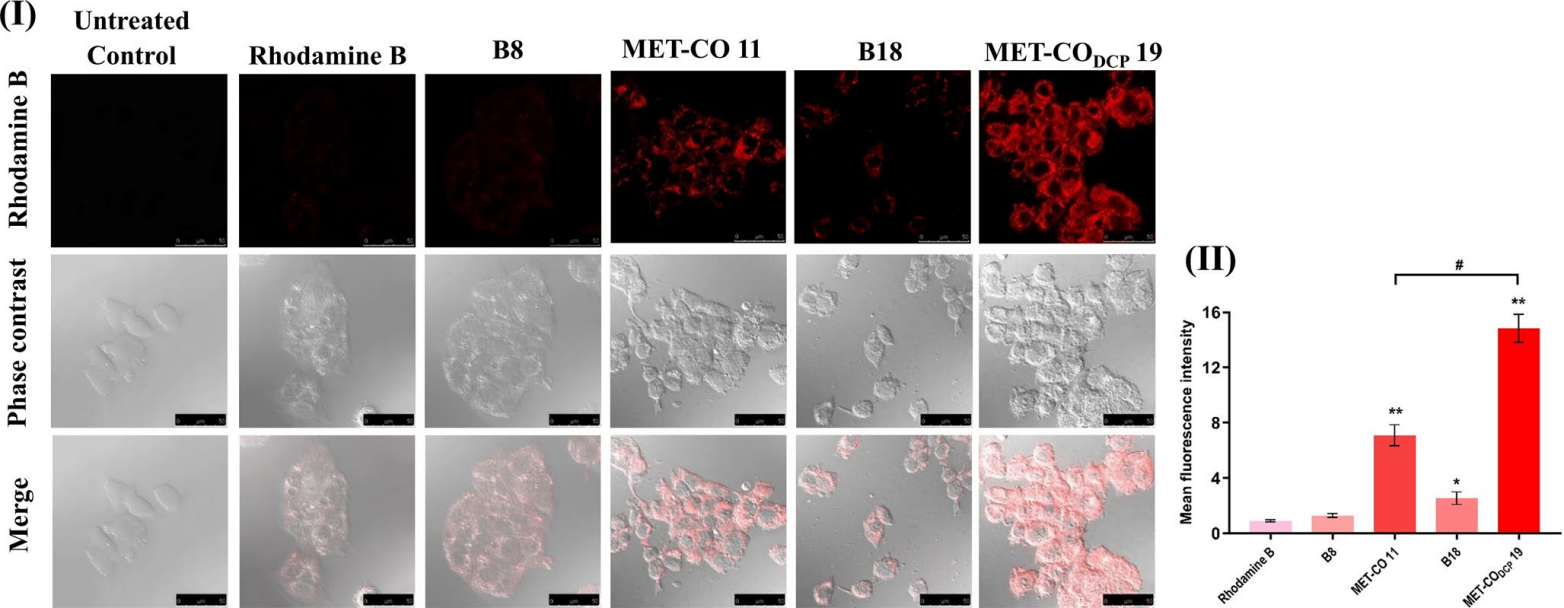
HepG2 cellular uptake of rhodamine-B dye and labelled MET-bridged nanocochleates. **(I)** CLSM images showing cellular uptake of the rhodamine-B dye, MET-CO 11, MET-CO_DCP_ 19, and their respective blank liposomes (B8 and B18), respectively after 1 h incubation. For all treatment groups, cells were incubated with the 24 h-IC_50_ of MET solution (53.93 ± 0.78 mM). Scale bar 50 μm. **(II)** Quantification of mean red fluorescence intensity of cellular uptake of rhodamine-B dye, MET-CO 11, and MET-CO_DCP_ 19 formulations and their respective blank counterparts (B8, B18) in HepG2 cells using confocal laser scanning microscope (CLSM) images and Image J software (version 1.52a). The data of bar graphs are presented as mean ± SD and statistically analyzed using one-way ANOVA followed by post-hoc test (Tukey), (*n* = 3). Statistical differences were significant (* *p* ≤ 0.05, ** *p* ≤ 0.0001 vs. Rhodamine B dye and # *p* ≤ 0.0001 vs. MET-CO 11)

CLSM images (Fig. 8 (I)) show higher significant internalization of MET-CO_DCP_ 19 in HepG2 cells (*p* ≤ 0.05) compared to MET and MET-CO 11 administered at doses equivalent to the 24 h-IC_50_ of MET solution (53.93 ± 0.78 mM). Confocal images (Fig. 8 (II)) depicted 2.1- and 16.5-fold higher red fluorescence from MET-CO_DCP_ 19 compared to MET-CO 11 or rhodamine-B dye alone, respectively. Cellular internalization could be ascribed to the fusion between the outer layer of nanocochleates and the cell membranes [54] and might have resulted in membrane reordering. Thereby, MET-bridged nanocochleates may be taken up by endocytosis and escape from the endocytic vesicle to deliver the encochleated MET into the cytosol of the target tumor cell in a controlled pattern. Overall, this result confirms the superior cellular internalization and cytosolic delivery from MET-CO_DCP_ 19.

The Bcl-2 class members play a key role in modulating the mitochondrial-dependent apoptotic pathway and are responsible for the negative regulation of cell apoptosis, thus promoting cell survival [24, 65]. MET-induced apoptosis has been attributed to the increased expression of the Bax/Bcl-2 ratio in HepG2 cells by activation of the AMPK/p53 pathway [66]. Conversely, the NANOG gene functions as a crucial transcription factor in regulating cancer cells and serves as a predictive marker in HCC [67]. In an AMPK pathway-independent manner, MET controls the expression of NANOG by inhibiting c-Jun N-terminal kinase (JNK) activity in HepG2 cells [68]. The JNK/NANOG signaling pathway plays a pivotal role in cell proliferation, programmed cell death, and the development and progression of various malignancies [69].

**Fig. 9:**
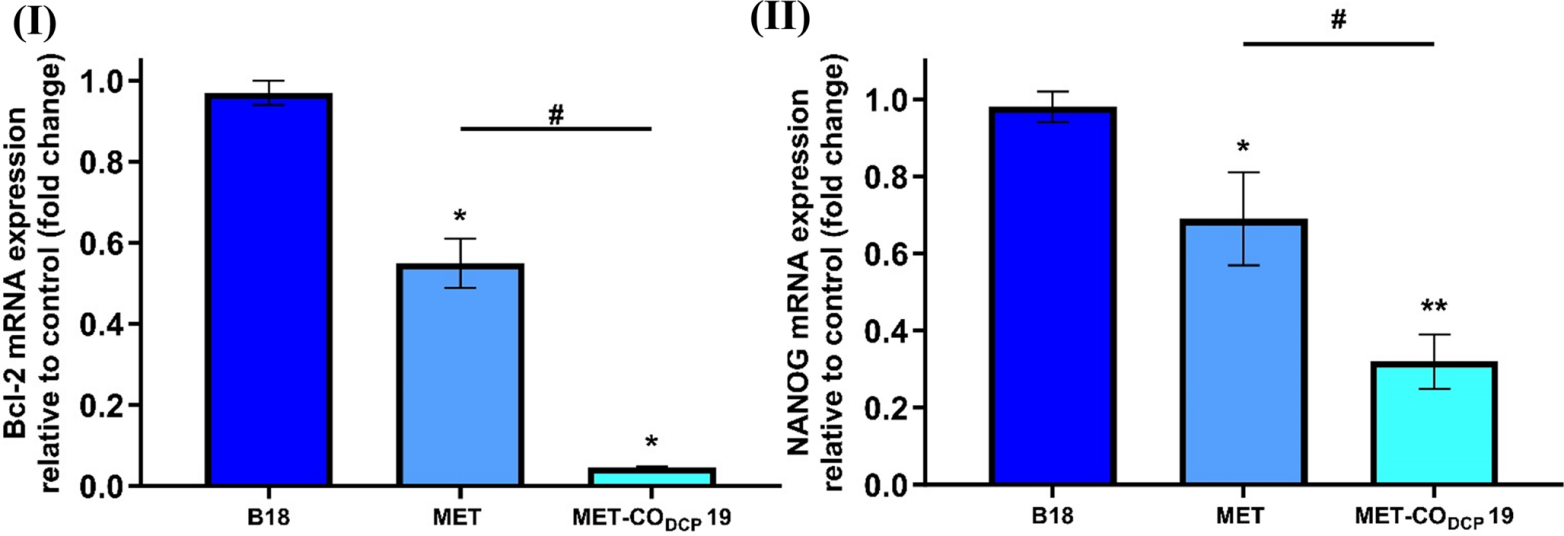
Suppression of anti-apoptotic, Bcl-2, and cancer stemness, NANOG, biomarker genes in HepG2 cells following treatment with MET-bridged nanocochleates. Expression levels of **(I)** Bcl-2 and **(II)** NANOG genes in HepG2 cells after treatment with the IC_50_ (24 h-treatment) of MET for 24 h in the following groups: MET, MET-CO_DCP_ 19 and its blank counterpart compared to the control untreated cells. Bcl-2 and NANOG expressions were measured by quantitative real-time PCR (qRT-PCR) and normalized against GAPDH levels as the housekeeping gene. All data are expressed as mean ± SD and statistically analyzed using one-way ANOVA followed by a post hoc test (Tukey’s), (*n* = 3). Statistical differences were significant for **(I)** (* *p* ≤ 0.0001 vs. the control group and # *p* ≤ 0.0001 vs. the MET group) and **(II)** (* *p* ≤ 0.01, ** *p* ≤ 0.0001 vs. the control group, and # *p* ≤ 0.01 vs. the MET group)

The qRT-PCR results (Fig. 9 (I)) demonstrated a significant downregulation of Bcl-2 expression in HepG2 cells after a 24-h incubation with MET-CO_DCP_ 19. This downregulation was 12-fold lower than that from MET solution (*p* ≤ 0.05). Similarly, MET-CO_DCP_ 19-treated HepG2 cells exhibited a significant downregulation in NANOG expression level (*p* ≤ 0.05), which was 2-fold lower than that from the MET solution (Fig. 9 (II)). There were no significant differences between the blank formulation (B18) and the control untreated group, as shown in Fig. 9 (I) and (II). This indicates that the placebo liposomal formulation and the constituting lipids had no impact on the target genes, and the downregulation of these genes was solely attributed to the enhanced anticancer efficacy of MET achieved through formulation as MET-bridged nanocochleates.

These data suggest that MET-CO_DCP_ 19 has the potential to enhance the anticancer effectiveness of MET in HepG2 cells by reducing the expression of anti-apoptotic (Bcl-2) and cancer stemness (NANOG) biomarker genes. This study is the first to propose lipid nanocochleates as a novel therapeutic nanoplatform for delivering MET in the treatment of HCC and to demonstrate the potential of MET-bridged nanocochleates to suppress Bcl-2 and NANOG expression levels in HepG2 cells.

## Conclusions and future directions

In this study, we present the successful development of novel MET-bridged nanocochleates. These nanocochleates were created by transforming blank PC/DCP-based anionic SUVs into self-assembled helix-shaped structures using a newly designed, simple, and reproducible direct bridging method. The resulting MET-CO_DCP_ 19 nanocochleates exhibited excellent physicochemical characteristics, including strong ionic coupling, high %EE, and product yield. Moreover, these nanocochleates demonstrated long shelf-life stability after lyophilization. One intriguing finding was that the MET-CO_DCP_ 19 nanocochleates controlled and prolonged the release of MET. This, combined with their lipophilicity, contributed to enhanced oral permeability, as demonstrated by independent Caco-2 transport and ex-vivo intestinal permeation models. Furthermore, when compared to commercial tablets or MET solution, oral administration of MET-CO_DCP_ 19 nanocochleates significantly enhanced the various pharmacokinetic parameters of MET. The nanocochleates also exhibited a prolonged half-life in the blood, resulting in enhanced oral bioavailability.

Appraising optimal preparation and storage conditions whilst emphasizing the enhanced oral permeability and absorption, the anticancer activity against HepG2 was profoundly scrutinized. Interestingly, MET-CO_DCP_ 19 demonstrated increased cellular uptake compared to MET-CO 11, leading to enhanced antiproliferative efficacy through an augmented apoptotic effect. This effect was supported by a significant knockdown of anti-apoptotic genes (Bcl-2) and cancer stemness biomarker genes (NANOG) in HepG2 cells. Overall, these promising results highlight the unique potential of MET-bridged nanocochleates as a nanoplatform for efficient oral delivery of MET in the management of hepatocellular carcinoma with lower dosing frequency, minimal side effects and higher patient compliance.

Forthcoming advanced studies are pivotal interrogates to better and deeply understand the exact underlying mechanism of MET-bridged nanocochleates’ interaction with the intestinal membrane and permeation through it along with intracellular tracking of its bridged payload. We further envisage that future comprehensive investigations will include the elucidation of signaling cascades and target protein expressions that may be involved in enhanced anticancer activity through MET encochleation in a liver tumor-bearing animal model. This will pave the way to better translate the distinctive features of the developed nano-self-assembly into clinical trials.

## Electronic supplementary material

Below is the link to the electronic supplementary material.

Supplementary Material 1

Supplementary Material 2

Supplementary Material 3

Supplementary Material 4

## Data Availability

All data generated or analysed during this study are included in this published article [and its supplementary information files].
