# Supplementary material for: Stable self-assembled oral metformin-bridged nanocochleates against hepatocellular carcinoma
Source: Drug Deliv Transl Res. 2024 Nov 13;15(6):2064–86. doi: 10.1007/s13346-024-01724-5 (PMC12037436; doi:10.1007/s13346-024-01724-5)
Supplement: Supplementary file 1 — Supplementary Material 1 [file 13346_2024_1724_MOESM1_ESM.docx]

**Stable Self-Assembled Oral Metformin-Bridged Nanocochleates against Hepatocellular Carcinoma**

*Mohamed G. El-Melegy^a^, Amal H. El-Kamel ^a^, Radwa A. Mehanna^b,c^, Ahmed Gaballah^d^, Hoda M. Eltaher^a, *^*

^a^ *Department of Pharmaceutics, Faculty of Pharmacy, Alexandria University, 21521, Alexandria, Egypt*

*^b^ Medical Physiology Department, Faculty of Medicine, Alexandria University, Alexandria, Egypt*

*^c^ Center of Excellence for Research in Regenerative Medicine and Applications CERRMA, Faculty of Medicine, Alexandria University, Alexandria, Egypt*

*^d^ Microbiology Department, Medical Research Institute, Alexandria University, 21561, Alexandria, Egypt*

^*^Corresponding Author

Email: [hoda.amin@alexpharmacy.edu.eg](mailto:hoda.amin@alexpharmacy.edu.eg)

The ORCID identification number for the corresponding author of this article can be found at: <https://orcid.org/0000-0001-5602-2696>

### Supplementary Information

### S1: High-performance liquid chromatography (HPLC) assay of MET

A reported validated HPLC method developed by Choi et al. was utilized for quantiﬁcation of MET with slight modiﬁcations. The HPLC instrument (Agilent Technologies-1260 Inﬁnity, Germany) was equipped with a UV-variable wavelength detector (G1314F) set at λ_max_ 232 nm, a reversed-phase C_18_ column (Agilent HC-C_18_ [4.6×250 mm], 5 μm particle size) and Agilent ChemStation^®^ software 32-bit version (revision B.02.01 SR1). A degassed 50:50 (v/v) mixture of 10 mM KH_2_PO_4_ buffer, pH 6, and acetonitrile, was utilized as an isocratic mobile phase. The mobile phase was run at a ﬂow rate of 1.0 mL/min at room temperature, and the injected sample volume was 20 µL. The analytical method was validated concerning linearity, speciﬁcity, precision, limits of detection and quantiﬁcation, and recovery.

The HPLC method was validated in terms of linearity, speciﬁcity, precision, limits of detection and quantiﬁcation, and recovery. MET was quantiﬁed from the standard calibration curve ﬁtting the equation (Y = 91347X - 2160), (R^2^ = 0.9996), covering a linearity range of 0.3–5 µg/mL. The lower limit of quantiﬁcation was 0.3 µg/mL, the intra-day and inter-day precision were less than 1.49%, while the % recoveries ranged from 98.88 to 102.09%.

### S2: Permeability Coefficient

The apparent permeability coefficient (P_app_) values of MET and the optimized formulations were then calculated according to **Equation 1**.

$P_{\mathrm{app}}= V_{R}*\frac{\mathrm{dC}}{\mathrm{dt}}*\frac{1}{A *C_{0}}$ **(Equation 1)**

Where,

- P_app_: Apparent permeability coefficient (cm/s)
- dc/dt: Cumulative concentration of drug (c) appearing in the basolateral chamber as a function of time (t) and was obtained from the slope of the linear portion of the concentration *vs.* time plot.
- A: Surface area of the monolayer filter
- C_0_: Initial concentration of drug in the apical chamber (mg/mL)
- V_R_: Volume of the basolateral chamber (mL)

**Table S1. Optimization of formulation variables of blank liposomes (B) and MET-bridged nanocochleates (MET-CO) using different preparation and loading techniques.**

| Formula code | Preparation  technique | MET addition technique | MET loading^^[[1]](#footnote-1)^^ (mg) | DCP loading^^[[2]](#footnote-2)^^ (mg) |
| --- | --- | --- | --- | --- |
| **B1** | **Thin film hydration** | --- | --- | --- |
| MET-CO 2 |  | Direct bridging | 50 |  |
| MET-CO 3 |  |  | 75 |  |
| MET-CO 4 |  |  | 100 |  |
| MET-CO 5 |  | Trapping | 50 |  |
| MET-CO 6 |  |  | 75 |  |
| MET-CO 7 |  |  | 100 |  |
| **B8** | **Ethanol injection** | --- | --- |  |
| MET-CO 9 |  | Direct bridging | 50 |  |
| MET-CO 10 |  |  | 75 |  |
| MET-CO 11 |  |  | 100 |  |
| MET-CO 12 |  | Trapping | 50 |  |
| MET-CO 13 |  |  | 75 |  |
| MET-CO 14 |  |  | 100 |  |
| **B15** |  | --- | --- | 10 |
| MET-CO_DCP_ 16 |  | Direct bridging | 100 |  |
| MET-CO_DCP_ 17 |  | Trapping |  |  |
| B18 |  | --- | --- | 20 |
| MET-CO_DCP_ 19 |  | Direct bridging | 100 |  |
| MET-CO_DCP_ 20 |  | Trapping |  |  |

**(A)**

**Metformin**

**MET-CO_DCP_ 19**

**physical mixture**

**MET-CO_DCP_ 19**

**formula**

**NH_2_**

**NH**

**(B)**


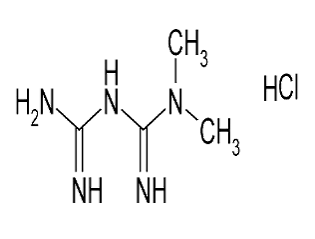

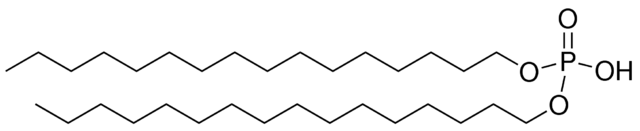

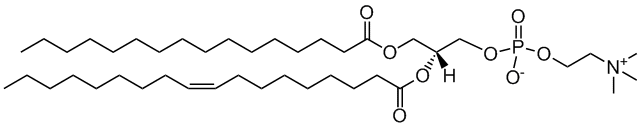


**Metformin**

**Egg PC**

**DCP**

**Fig. S1. (A)** Close-up on the FTIR region corresponding to the stretching band of amino groups (-NH_2_ and -NH). (**B)** schematic diagram showing ionic coupling between the negatively charged -OH groups of the lipid excipients (Lipoid^®^ E80 and DCP) and the positively charged -NH groups of Metformin HCl. The red dashed line refers to ionic interaction among functional groups.

**Abbreviations:** MET-CO, Metformin-bridged nanocochleates; PC, phosphatidyl choline; DCP, dicetyl phosphate.

**Table S2.** Fitting *in-vitro* release profiles of MET solution, MET-CO 11, MET-CO 14*,* MET-CO_DCP_ 19, and MET-CO_DCP_ 20 to different release kinetic models.

| Samples | Kinetic model | | | | | |
| --- | --- | --- | --- | --- | --- | --- |
|  | **First-order with T_lag_ and F_max_** | | **Korsmeyer-Peppas with T_lag_** | | **Weibull** | |
|  | Fitting equation  [ln(1- F/F_max_) = - k_1_*t + k_1_*T_lag_] | R^2^ | Fitting equation  [ln(F) = ln(k_KP_) + n*ln(t - T_lag_)] | R^2^ | Fitting equation  [F = 100*{1 - Exp[- (t^β^) / α]}] | R^2^ |
| MET solution | ln(1- F/99.989) = -4.525*t+4.525*-0.177 | 0.9849 | ln(F) = ln(94.732 )+0.03*ln(t-0.083) | 0.7897 | F = 100*{1-Exp[-(t^0.532^)/0.234]} | 0.9654 |
| MET-CO 11 | ln(1- F/73.515) = -2.591*t+2.591*-0.115 | 0.9733 | ln(F) = ln(61.791)+0.094*ln(t-0.083) | 0.9176 | F = 100*{1-Exp[-(t^0.233^)/1.108]} | 0.8539 |
| MET-CO_DCP_ 19 | ln(1- F/74.962) = -3.728*t+3.728*-0.043 | 0.9627 | ln(F) = ln(65.615)+0.078*ln(t-0.083) | 0.9449 | F = 100*{1-Exp[-(t^0.231^)/1.021]} | 0.7932 |
| MET-CO 14 | ln(1- F/66.078) = -3.295*t+3.295*-0.183 | 0.8404 | ln(F) = ln(59.977)+0.061*ln(t-0.083) | 0.9727 | F = 100*{1-Exp[-(t^0.146^)/1.160]} | 0.8408 |
| MET-CO_DCP_ 20 | ln(1- F/74.705) = -4.236*t+4.236*-0.131 | 0.9473 | ln(F) = ln(69.208)+0.041*ln(t-0.083) | 0.9603 | F = 100*{1-Exp[-(t^0.145^)/0.902]} | 0.7813 |

**Table S3.** Short-term stability study of the optimized formulations of MET-bridged nanocochleates (MET-CO 11 and MET-CO_DCP_ 19) at 4 ± 2 ℃ and 25% RH for up to one week. Data are expressed as mean ± SD and statistically analyzed using one-way ANOVA followed by Post-Hoc test (Tukey), (n = 3). No statistically significant differences (p ≤ 0.05) were observed within 7 days.

| Time course | | % Entrapment efficiency (EE) | |
| --- | --- | --- | --- |
|  |  | **MET-CO 11 formula** | **MET-CO_DCP_ 19 formula** |
| Fresh (zero-time) | | 54.86 ± 2.49 | 76.83 ± 1.68 |
| Post Formulation | 6 h | 53.11 ± 2.15 | 75.98 ± 1.57 |
|  | 2 d | 53.64 ± 2.36 | 76.09 ± 1.71 |
|  | 3 d | 53.05 ± 1.9 | 75.96 ± 1.32 |
|  | 4 d | 52.87 ± 1.48 | 75.8 ± 1.62 |
|  | 5 d | 52.47 ± 1.69 | 75.73 ± 1.5 |
|  | 6 d | 52.1 ± 1.34 | 75.48 ± 1.4 |
|  | 7 d | 51.97 ± 1.42 | 75.18 ± 1.65 |


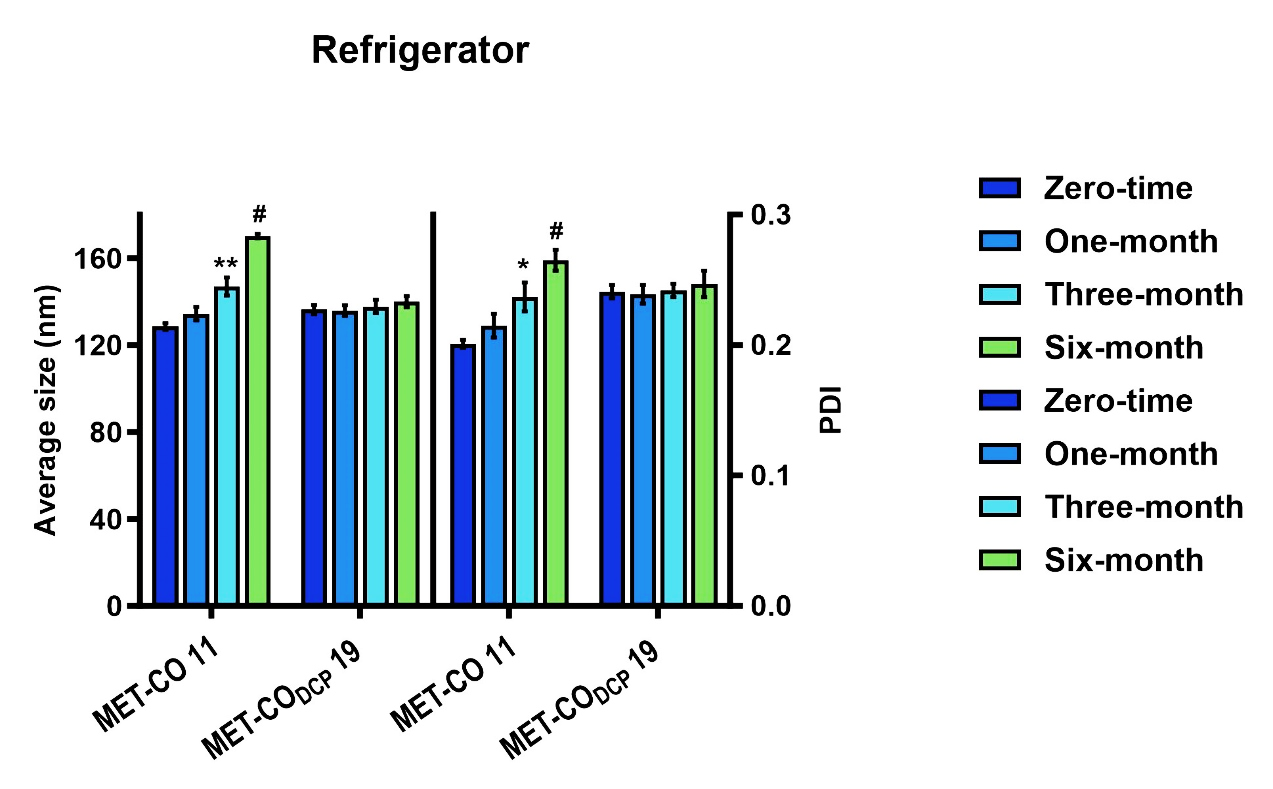

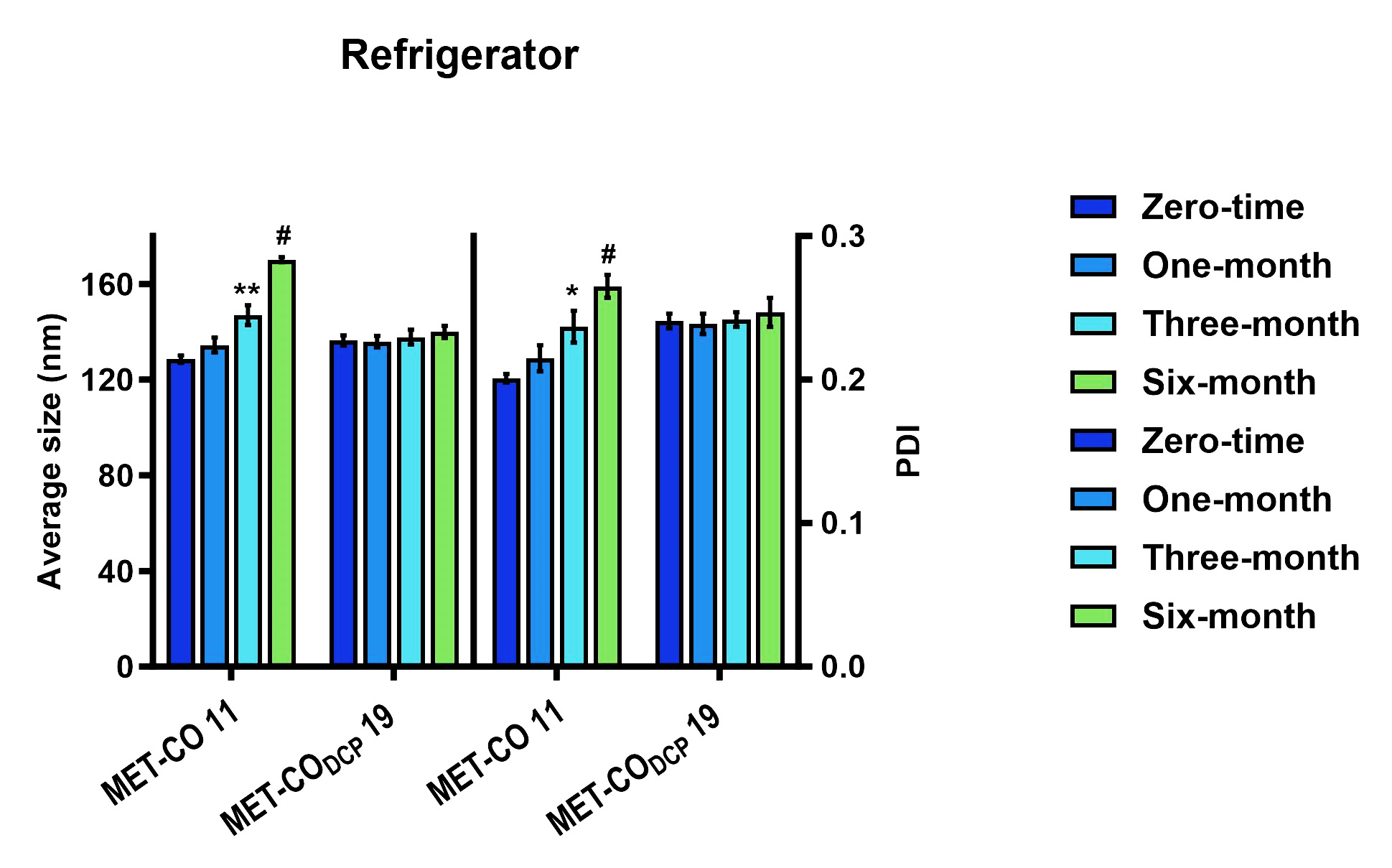

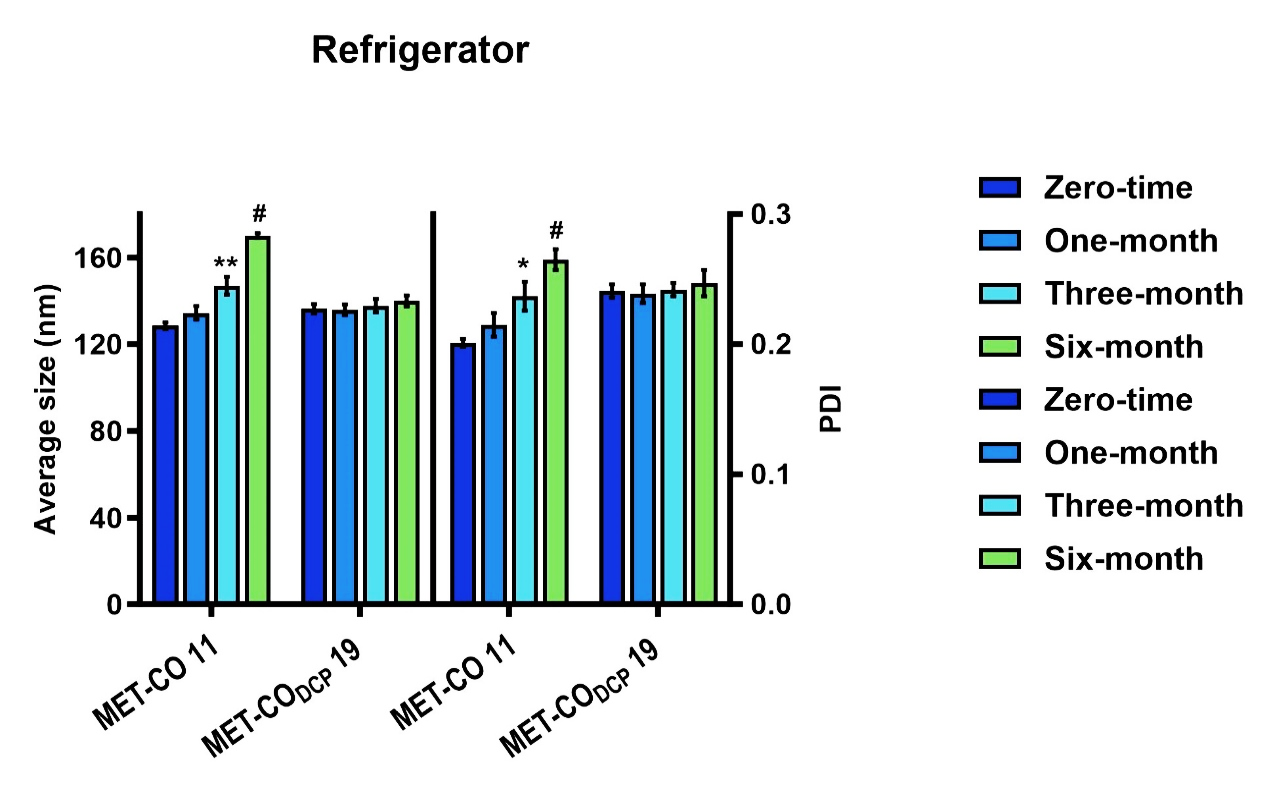

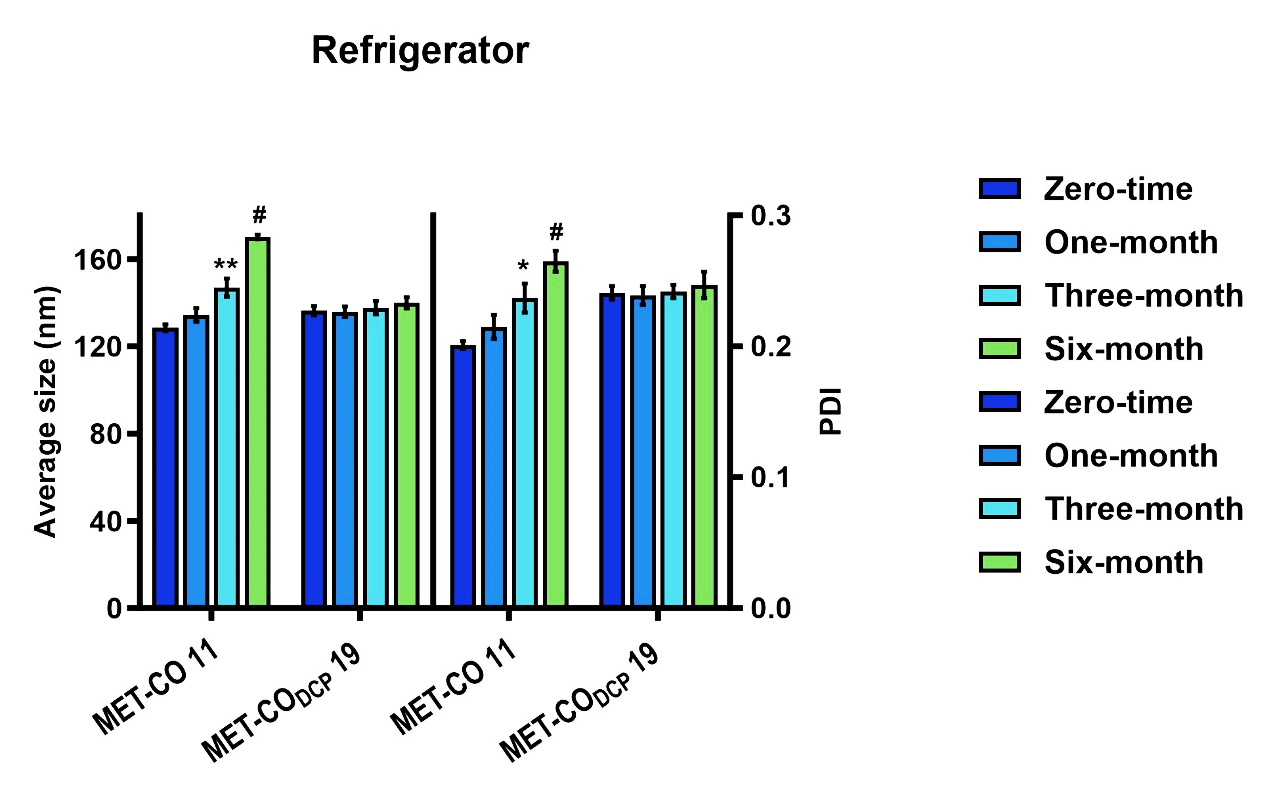

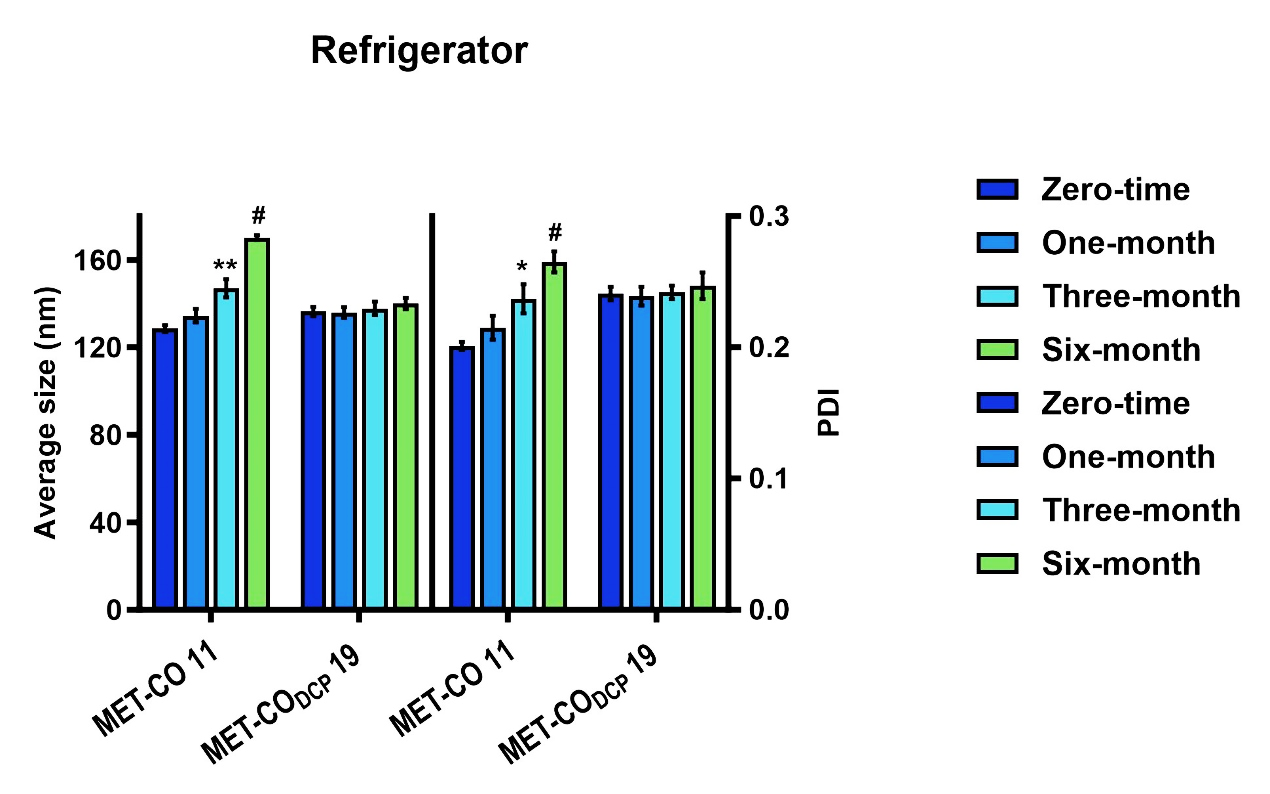

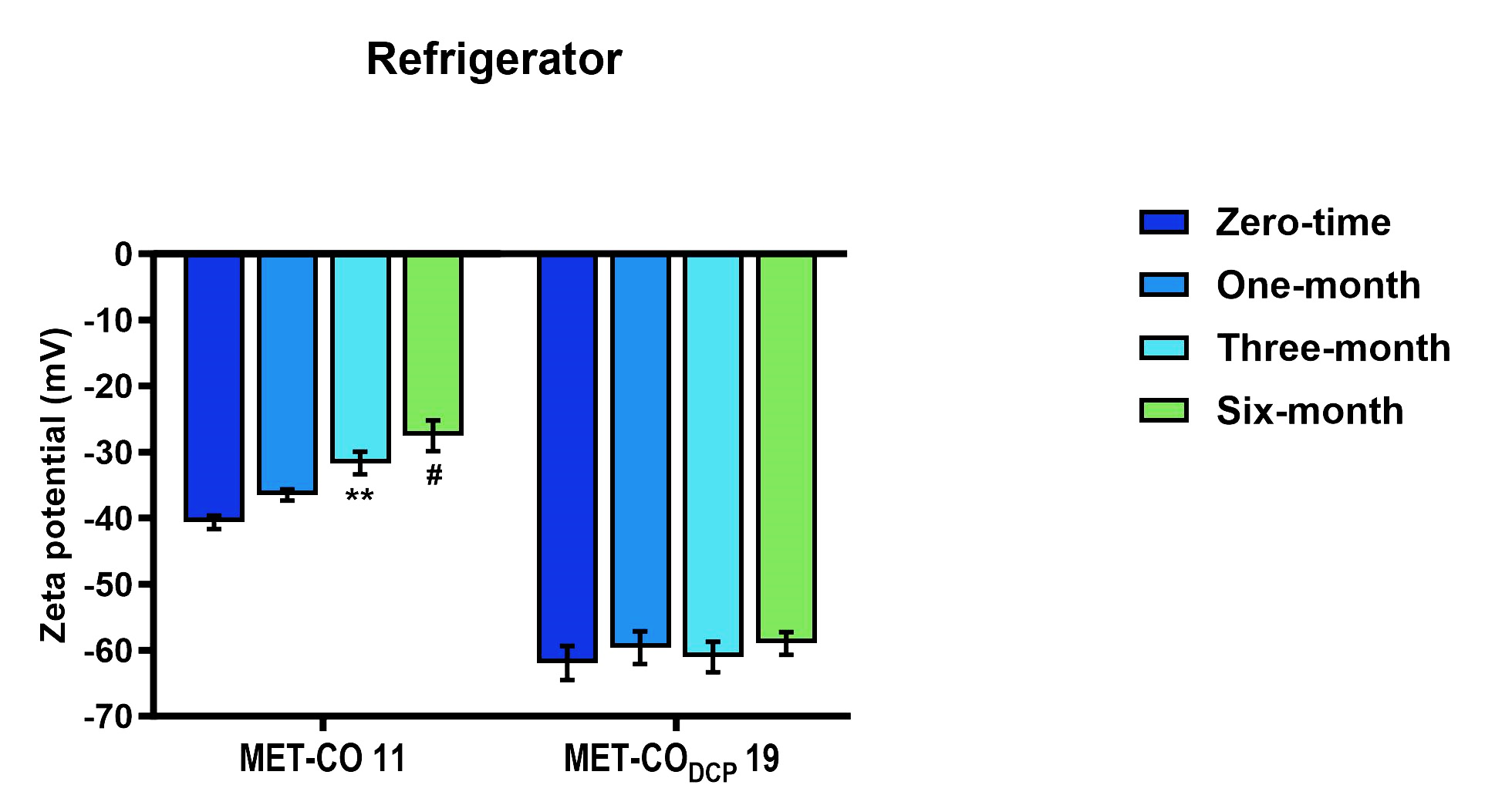

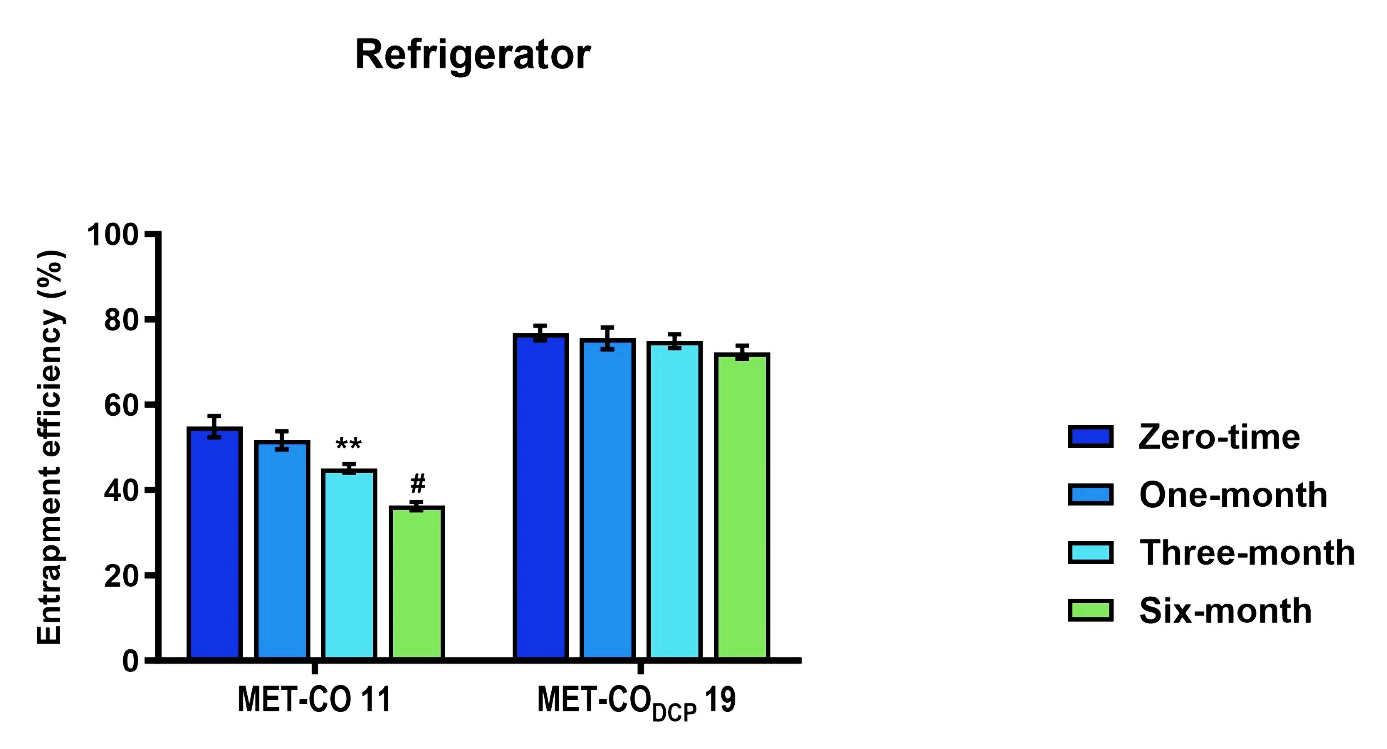


**Fig. S2.** **Long-term stability study of** **the optimized formulations of MET-bridged nanocochleates (MET-CO 11 and MET-CO_DCP_ 19) after 6 months of storage in the form of lyophilized powder at** **4 ± 2 ℃ and 25% RH.** Data are presented as mean ± SD and statistically analyzed using one-way ANOVA followed by post-hoc test (Tukey), (*n* = 3). (* p ≤ 0.01, ** p ≤ 0.001, and # p ≤ 0.0001 *vs.* zero-time).


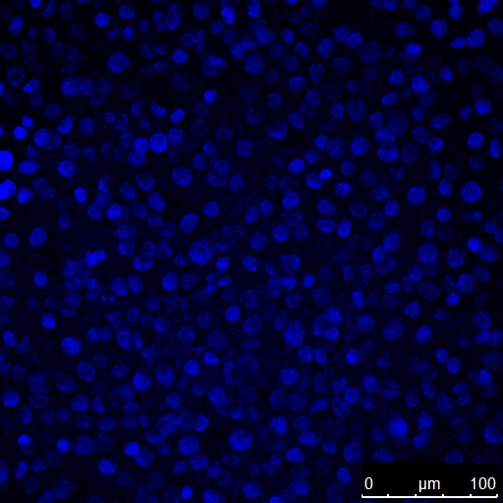


**Fig. S3.** Caco-2 cells cultured on the apical compartment of the Transwell^®^ inserts formed a monolayer shown by the nuclear fluorescent Hoechst 33342 stain (blue fluorescence) visualized under confocal laser scanning microscope (CLSM) prior to the transport experiment (scale bar 100 µm).

1. ^1^Metformin loading per 70 mg of Lipoid^®^ E80 and 30 mg of cholesterol.

   ^2^Dicetyl phosphate loading per 70 mg of Lipoid^®^ E80 and 30 mg of cholesterol. [↑](#footnote-ref-1)
2. [↑](#footnote-ref-2)
