# Supplementary figures and images for: Stable self-assembled oral metformin-bridged nanocochleates against hepatocellular carcinoma
Source: Drug Deliv Transl Res. 2024 Nov 13;15(6):2064–86. doi: 10.1007/s13346-024-01724-5 (PMC12037436; doi:10.1007/s13346-024-01724-5)

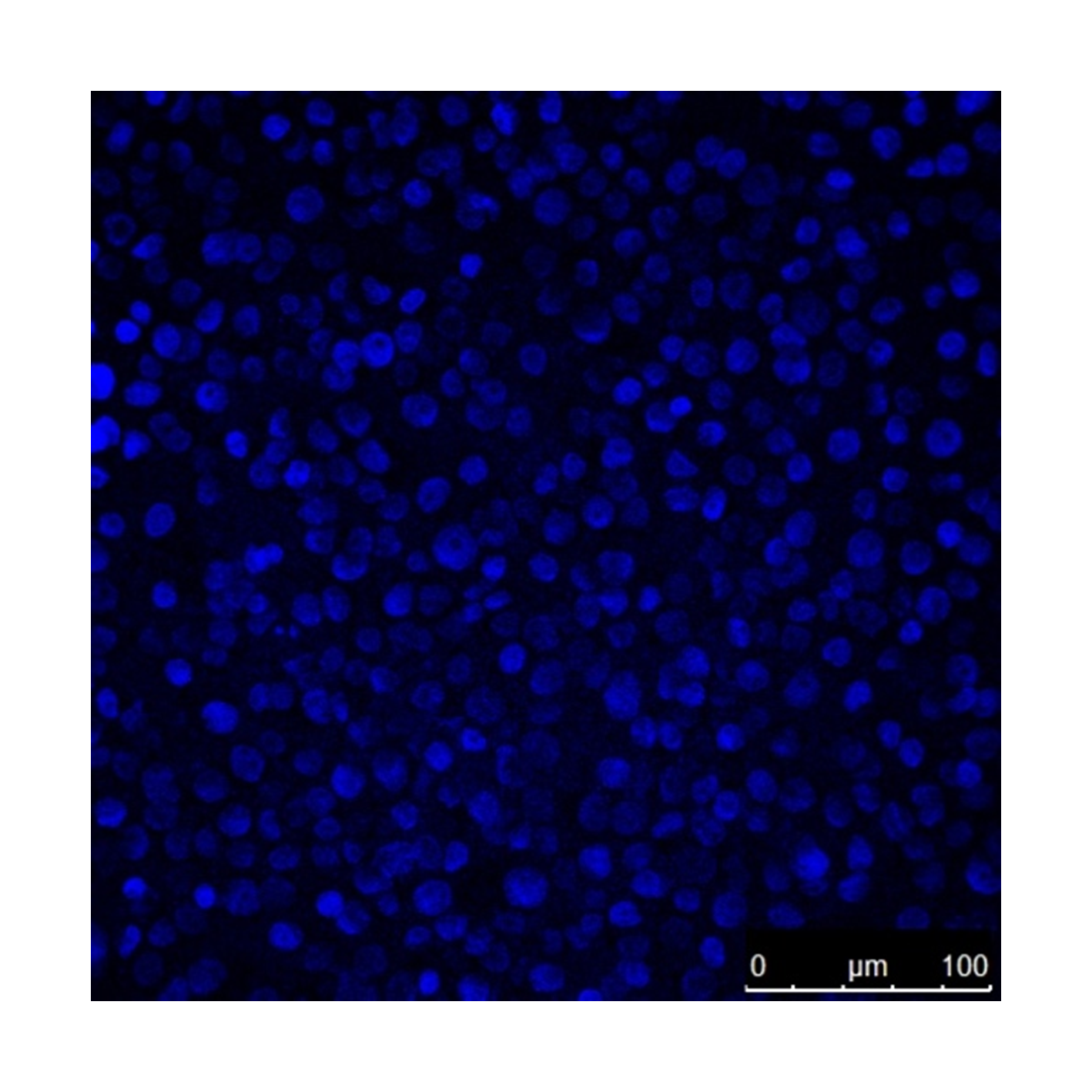

Supplement: Supplementary file 2 — Supplementary Material 2 [file 13346_2024_1724_MOESM2_ESM.tif]

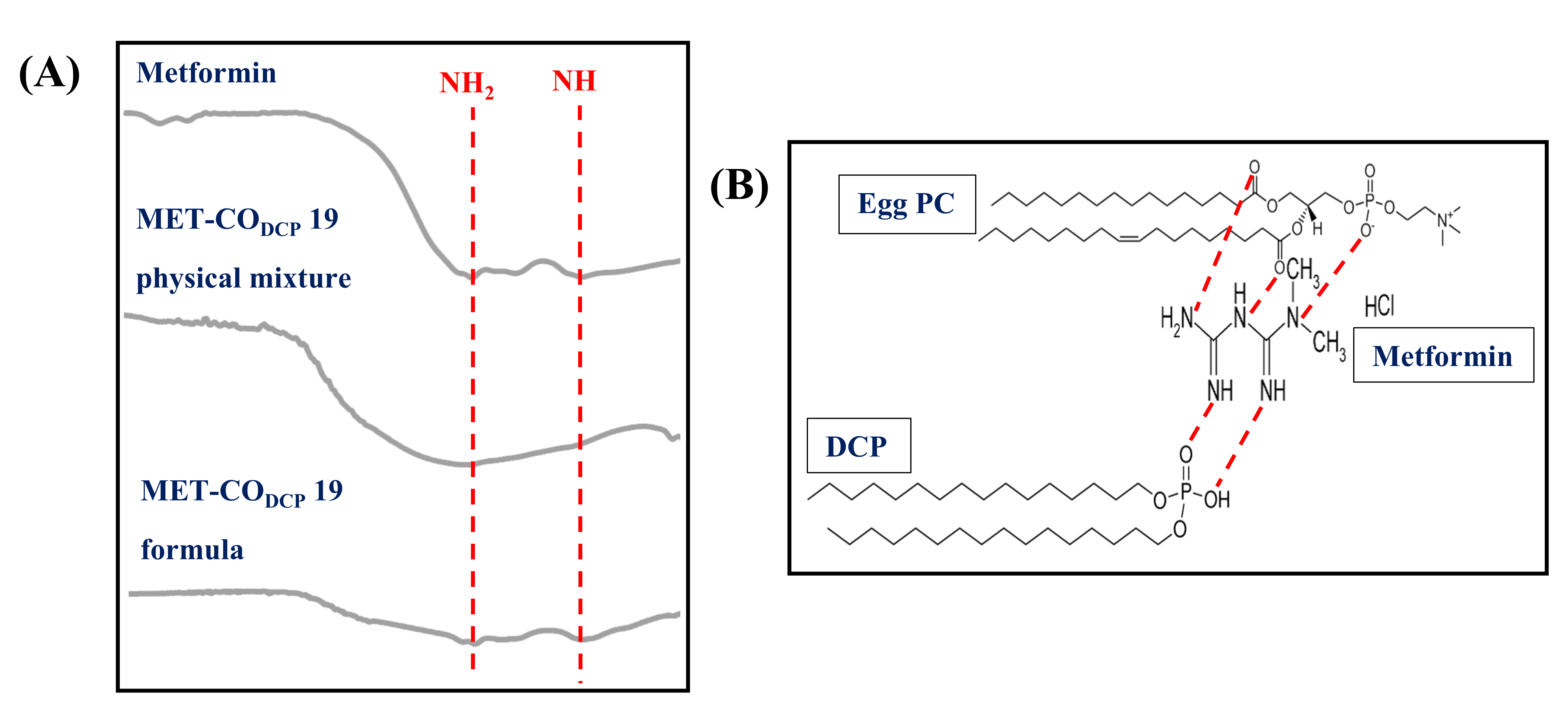

Supplement: Supplementary file 3 — Supplementary Material 3 [file 13346_2024_1724_MOESM3_ESM.tif]

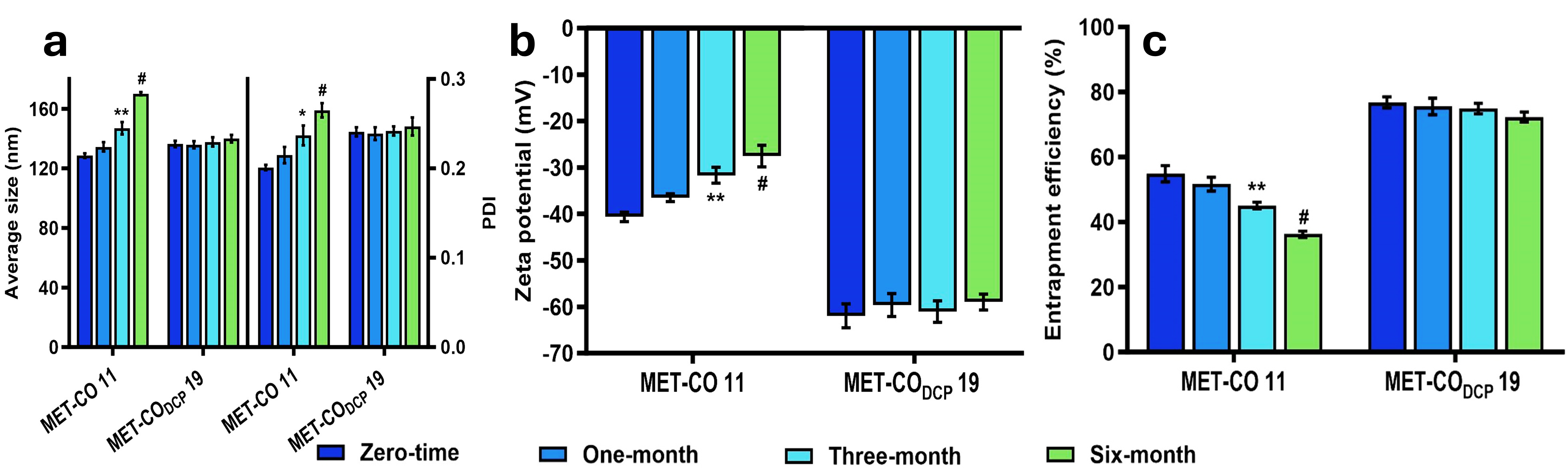

Supplement: Supplementary file 4 — Supplementary Material 4 [file 13346_2024_1724_MOESM4_ESM.tif]
